# Predictors of critical care, mechanical ventilation, and mortality among hospitalized patients with COVID-19 in an electronic health record database

**DOI:** 10.1186/s12879-022-07383-6

**Published:** 2022-04-29

**Authors:** Andrea K. Chomistek, Caihua Liang, Michael C. Doherty, C. Robin Clifford, Rachel P. Ogilvie, Robert V. Gately, Jennifer N. Song, Cheryl Enger, Nancy D. Lin, Florence T. Wang, John D. Seeger

**Affiliations:** 1 Optum, 1325 Boylston Street, 11th Floor, Boston, MA 02215 USA; 2 IQVIA, Plymouth Meeting, PA USA

**Keywords:** Coronavirus, SARS-CoV-2, Risk factors, Death, Biomarkers, Treatments

## Abstract

**Background:**

There are limited data on risk factors for serious outcomes and death from COVID-19 among patients representative of the U.S. population. The objective of this study was to determine risk factors for critical care, ventilation, and death among hospitalized patients with COVID-19.

**Methods:**

This was a cohort study using data from Optum’s longitudinal COVID-19 electronic health record database derived from a network of healthcare provider organizations across the US. The study included patients with confirmed COVID-19 (presence of ICD-10-CM code U07.1 and/or positive SARS-CoV-2 test) between January 2020 and November 2020. Patient characteristics and clinical variables at start of hospitalization were evaluated for their association with subsequent serious outcomes (critical care, mechanical ventilation, and death) using odds ratios (OR) and 95% confidence intervals (CI) from logistic regression, adjusted for demographic variables.

**Results:**

Among 56,996 hospitalized COVID-19 patients (49.5% male and 72.4% ≥ 50 years), 11,967 received critical care, 9136 received mechanical ventilation, and 8526 died. The median duration of hospitalization was 6 days (IQR: 4, 11), and this was longer among patients that experienced an outcome: 11 days (IQR: 6, 19) for critical care, 15 days (IQR: 8, 24) for mechanical ventilation, and 10 days (IQR: 5, 17) for death. Dyspnea and hypoxemia were the most prevalent symptoms and both were associated with serious outcomes in adjusted models. Additionally, temperature, C-reactive protein, ferritin, lactate dehydrogenase, D-dimer, and oxygen saturation measured during hospitalization were predictors of serious outcomes as were several in-hospital diagnoses. The strongest associations were observed for acute respiratory failure (critical care: OR, 6.30; 95% CI, 5.99–6.63; ventilation: OR, 8.55; 95% CI, 8.02–9.11; death: OR, 3.36; 95% CI, 3.17–3.55) and sepsis (critical care: OR, 4.59; 95% CI, 4.39–4.81; ventilation: OR, 5.26; 95% CI, 5.00–5.53; death: OR, 4.14; 95% CI, 3.92–4.38). Treatment with angiotensin-converting enzyme inhibitors/angiotensin receptor blockers during hospitalization were inversely associated with death (OR, 0.57; 95% CI, 0.54–0.61).

**Conclusions:**

We identified several clinical characteristics associated with receipt of critical care, mechanical ventilation, and death among COVID-19 patients. Future studies into the mechanisms that lead to severe COVID-19 disease are warranted.

**Supplementary Information:**

The online version contains supplementary material available at 10.1186/s12879-022-07383-6.

## Introduction

Since coronavirus disease 2019 (COVID-19) first emerged in China in December 2019, there have been over 270 million confirmed cases and 5.3 million deaths worldwide due to COVID-19 as of 15 December 2021, according to the World Health Organization [1]. The United States (US) leads the world in cases (49.8 million) and deaths (792,371) from COVID-19 and these numbers are expected to continue to rise through the start of 2022.

There is significant heterogeneity in the clinical presentation of COVID-19 infection, ranging from patients who are asymptomatic to those with severe disease [2–4]. It is important to determine predictors of serious outcomes as patients may decline rapidly after initially presenting with mild symptoms [5]. Identifying predictors of serious outcomes may enable clinicians to deliver appropriate care to patients early as well as inform interventions to reduce risk of death [6].

The serious outcomes of COVID-19 (e.g., intensive care unit admission, receipt of mechanical ventilation, death) and their preceding risk factors have been identified previously [7–10]. Common factors associated with progression to serious disease include age, male sex, obesity, and comorbid diseases, including diabetes and renal disease. Additionally, it has been recognized that biomarkers, such as C-reactive protein (CRP) and D-dimer, may be associated with serious outcomes. However, studies from early in the pandemic were small and sought to identify the strongest predictors of serious disease and death from a broad set of variables. Further, some of these studies were hospital-based case series and may not be representative of all patients hospitalized with COVID-19 in the United States.

The purpose of this study was to apply an exploratory, data-driven approach to the identification of potential risk factors for serious outcomes among patients with COVID-19 in order to inform clinicians and researchers of characteristics that may be integral to identifying high risk patients. Thus, the objective was to determine demographic and clinical predictors associated with critical care, mechanical ventilation, and death among hospitalized COVID-19 patients in a large electronic health record (EHR) database that is representative of a geographically diverse U.S. population.

## Methods

### Study design and study population

This was a retrospective cohort study that included patients confirmed with COVID-19 infection between January 2020 and November 2020. Among these patients, a subset of patients hospitalized with COVID-19 was identified from those with an inpatient health care encounter. Confirmation of COVID-19 infection was based on presence of a specific International Classification of Diseases, 10^th^ Revision, Clinical Modification (ICD-10-CM) diagnosis code (U07.1) and/or a positive severe acute respiratory syndrome coronavirus 2 (SARS-CoV-2) viral test. The date of confirmed infection was the earlier of the date of diagnosis or the date of a positive test. The cohort entry date was the earliest date that the patient met both of the following criteria: confirmed COVID-19 infection and admission to the hospital. For patients who were hospitalized prior to contracting COVID-19, the date of cohort entry was the date of confirmed infection. For patients who were confirmed to have COVID-19 before they were admitted to the hospital, the date of cohort entry was date of hospital admission. This approach for assigning cohort entry date allows for the description of clinical characteristics at the time patients were first hospitalized with COVID-19. For clinical characteristics other than death, patients were followed from cohort entry to discharge or 30 days after cohort entry, whichever came first. Deaths were identified in all follow-up available, including during and after hospitalization.

### Data source

Patients were identified from Optum’s longitudinal COVID-19 EHR database derived from a network of healthcare provider organizations across the U.S. The COVID-19 EHR database consists of a subset of patients from Optum’s EHR database, which represents a geographically diverse U.S. patient population with more than 85 million patients from 2007 through 2019. Optum’s EHR database includes data collected from tens of thousands of providers and hundreds of hospitals representing more than 60 electronic medical record (EMR)-based provider/hospital networks across the U.S. This database incorporates clinical and medical administrative data from both inpatient and ambulatory EMRs, practice management systems, and numerous other internal systems. Information is processed from across the continuum of care, including acute inpatient stays and outpatient visits. The COVID-19 data captures diagnostics specific to the COVID-19 patient during initial presentation at hospital admission, acute illness, and convalescence with over 500 mapped labs and bedside observations, including COVID-19 specific testing. The data are incorporated into the underlying database on a biweekly basis, allowing for near real-time analysis and assessment of the COVID-19 clinical landscape. The database is certified as de-identified by an independent statistical expert following Health Insurance Portability and Accountability Act statistical de-identification rules.

### Ascertainment of covariates

Demographic characteristics were assessed on the date of cohort entry. Comorbidities and medication use were assessed in the 21 days prior to cohort entry. Comorbidities were identified by ICD-10-CM diagnosis code with a diagnosis status of “history of” and medications were mapped according to the Anatomical Therapeutic Chemical (ATC) classification scheme.

Additionally, vital signs, laboratory results, symptoms, diagnoses, and treatments during hospitalization were assessed. For vital signs and laboratory results, the first measurement on or after the date of cohort entry was selected if the patient had multiple measurements. Symptoms and diagnoses were identified by ICD-10-CM diagnosis codes.

### Identification of outcomes

The primary outcomes of interest were receipt of critical care, mechanical ventilation, and death. Patients were classified according to the presence or absence of an outcome, and outcome groups were not mutually exclusive.

Receipt of critical care was identified using Current Procedural Terminology, 4th Edition (CPT-4) codes. Receipt of mechanical ventilation was identified by CPT-4 and International Classification of Diseases, 10th Revision, Procedural Coding System (ICD-10-PCS) codes and included intubation, mechanical ventilation, and extracorporeal membrane oxygenation (ECMO). Receipt of critical care and mechanical ventilation were ascertained during hospitalization.

Death was ascertained via linkage to the Social Security Administration’s Death Master File and/or the presence of a death indicator in the structured EHR data within all data available during and after hospitalization.

### Statistical analysis

All analyses were conducted using SAS version 9.4 (SAS Institute Inc, Cary, NC). Baseline characteristics were examined overall and according to outcome (critical care, mechanical ventilation, and death). Categorical variables were summarized using frequency and percent while continuous variables were summarized using median and interquartile range (IQR).

For the association analyses, vital signs and laboratory values were transformed into categorical variables. Dichotomous variables were created based on clinically-relevant cutpoints. Additionally, categories based on quintiles were generated to examine the shape of the dose–response relationship of vital signs and laboratory values with each outcome. Quintiles were determined based on the distribution of each biomarker among hospitalized patients overall. Tests for linear trend were computed by using the medians of the quintiles modeled as a continuous variable.

Logistic regression models were used to estimate unadjusted and adjusted odds ratios (OR) and corresponding 95% confidence intervals (CI) for associations between the covariates and each outcome. Adjusted models included age, gender, region, race, and week of cohort entry. Week of cohort entry was included as a covariate to adjust for any potential changes in patient characteristics or treatments over time.

## Results

### Descriptive analyses

**Fig. 1:**
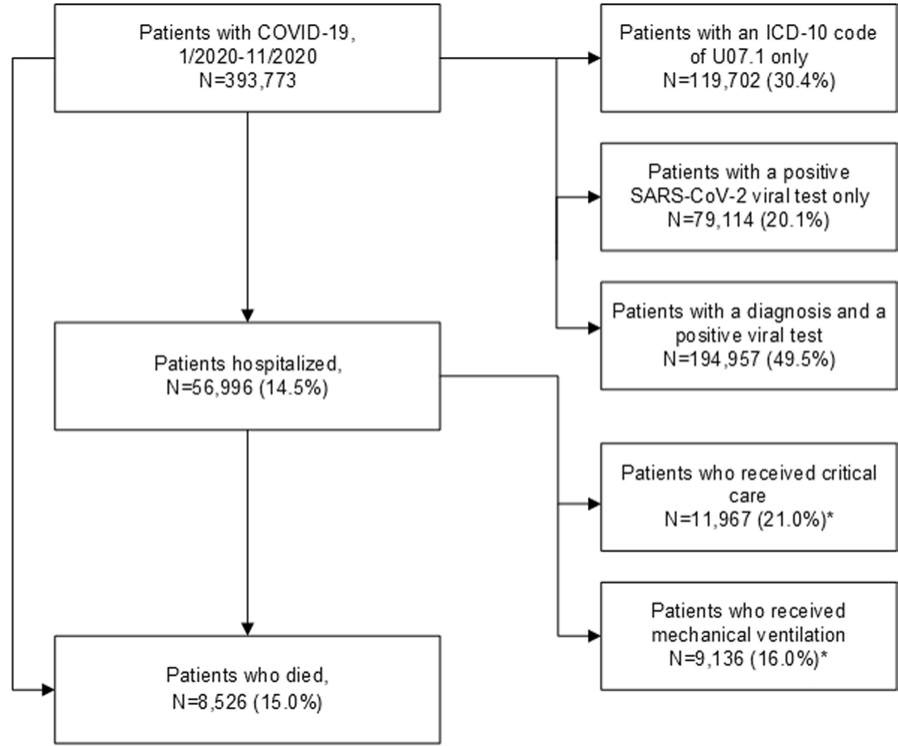
Flowchart of hospitalized COVID-19 patients: the Optum COVID-19 EHR Database, January–November 2020. *Patients who received critical care and mechanical ventilation were counted in both categories

A total of 56,996 hospitalized patients with COVID-19 between January 2020 and November 2020 were identified (Fig. 1). Of these, 11,967 (21.0%) received critical care, 9136 (16.0%) received mechanical ventilation, and 8526 (15.0%) died. Table 1 shows the demographics, comorbidities, and patient-reported medications at baseline overall and according to outcome. The majority of hospitalized patients were aged 50 years and older (72.4%), Caucasian (58.8%), and from the Midwest or Northeast (38.0% and 29.0%, respectively); this was also observed for each outcome. Females comprised 50.5% of hospitalized patients, but males comprised the majority of patients experiencing each outcome (58.8% for critical care, 60.4% for mechanical ventilation, and 57.4% for death). Prior to the cohort entry date, 76.7% of patients received care in the emergency department, while fewer patients received care in the outpatient or inpatient settings. Confirmation of COVID-19 infection was based on both a positive SARS-CoV-2 viral test and presence of a U07.1 diagnosis code for 70.2% of hospitalized patients, a U07.1 diagnosis code only for 26.0% of patients, and a positive SARS-CoV-2 viral test only for 3.8% of patients. The largest proportion of patients were admitted to the hospital in April 2020 (18.3%), followed by November 2020 (17.8%).

**Table 1:** Baseline characteristics among hospitalized COVID-19 patients, overall and by outcome

	Overall		Critical Care		Mechanical Ventilation		Death	
	N	%	N	%	N	%	N	%
Total patients	56,996	100.0	11,967	100.0	9136	100.0	8526	100.0
Age								
< 10	396	0.7	82	0.7	29	0.3	7	0.1
10–19	671	1.2	135	1.1	46	0.5	1	0.0
20–29	3610	6.3	293	2.4	175	1.9	37	0.4
30–39	5123	9.0	615	5.1	380	4.2	110	1.3
40–49	5889	10.3	1,113	9.3	794	8.7	285	3.3
50–59	9486	16.6	2,125	17.8	1605	17.6	770	9.0
60–69	11,871	20.8	2,926	24.5	2552	27.9	1658	19.4
70–79	10,396	18.2	2,621	21.9	2137	23.4	2257	26.5
80 +	9554	16.8	2,057	17.2	1418	15.5	3401	39.9
Gender								
Female	28,782	50.5	4925	41.2	3620	39.6	3636	42.6
Male	28,214	49.5	7042	58.8	5516	60.4	4890	57.4
Race								
African American	11,694	20.5	2282	19.1	1873	20.5	1529	17.9
Asian	1336	2.3	329	2.7	250	2.7	206	2.4
Caucasian	33,524	58.8	7125	59.5	5227	57.2	5536	64.9
Other/Unknown	10,442	18.3	2231	18.6	1786	19.5	1255	14.7
Ethnicity								
Hispanic	8497	14.9	1711	14.3	1271	13.9	814	9.5
Not Hispanic	42,881	75.2	9079	75.9	6910	75.6	6908	81.0
Unknown	5618	9.9	1177	9.8	955	10.5	804	9.4
Region								
Midwest	21,647	38.0	4979	41.6	3851	42.2	2995	35.1
Northeast	16,548	29.0	3621	30.3	2516	27.5	2616	30.7
South	12,654	22.2	2094	17.5	1583	17.3	2114	24.8
West	4378	7.7	947	7.9	949	10.4	560	6.6
Other/Unknown	1769	3.1	326	2.7	237	2.6	241	2.8
Care settings prior to admission date								
Inpatient	4684	8.2	747	6.2	736	8.1	945	11.1
Outpatient	10,479	18.4	2121	17.7	1554	17.0	1253	14.7
Emergency department	43,724	76.7	9819	82.1	7615	83.4	7194	84.4
Insurance type on admission date								
Multiple	16,266	28.5	3790	31.7	2831	31.0	3274	38.4
Commercial	15,775	27.7	2916	24.4	2193	24.0	1134	13.3
Medicare	12,364	21.7	2854	23.8	2258	24.7	2888	33.9
Medicaid	5111	9.0	1107	9.3	801	8.8	354	4.2
Other payer type	2936	5.2	483	4.0	364	4.0	275	3.2
Uninsured	973	1.7	156	1.3	109	1.2	68	0.8
Unknown	3571	6.3	661	5.5	580	6.3	533	6.3
Confirmatory event								
Positive SARS-CoV-2 viral test only	2166	3.8	258	2.2	215	2.4	318	3.7
U07.1 diagnosis code only	14,822	26.0	3027	25.3	2367	25.9	1989	23.3
Positive SARS-CoV-2 viral test and U07.1 diagnosis code	40,008	70.2	8682	72.5	6554	71.7	6219	72.9
Month of cohort entry								
January 2020 through March 2020	4497	7.9	1389	11.6	1397	15.3	1040	12.2
April 2020	10,422	18.3	2924	24.4	2205	24.1	2360	27.7
May 2020	5,859	10.3	1472	12.3	994	10.9	1028	12.1
June 2020	4611	8.1	908	7.6	594	6.5	593	7.0
July 2020	6082	10.7	1136	9.5	840	9.2	916	10.7
August 2020	5013	8.8	856	7.2	644	7.0	721	8.5
September 2020	4318	7.6	824	6.9	575	6.3	623	7.3
October 2020	6028	10.6	1102	9.2	858	9.4	776	9.1
November 2020	10,166	17.8	1356	11.3	1029	11.3	469	5.5
Comorbidities								
Diabetes	16,826	29.5	4851	40.5	3918	42.9	3375	39.6
Obesity	13,544	23.8	3861	32.3	3183	34.8	1893	22.2
Pulmonary disease								
COPD	5830	10.2	1,812	15.1	1582	17.3	1498	17.6
Asthma	4948	8.7	1,121	9.4	916	10.0	510	6.0
Cardiovascular disease								
Hypertension	23,229	40.8	5855	48.9	4495	49.2	4029	47.3
Coronary artery disease	8407	14.8	2477	20.7	1986	21.7	2228	26.1
Congestive heart failure	7242	12.7	2425	20.3	2058	22.5	2246	26.3
Kidney disease*	17,694	31.0	6103	51.0	5110	55.9	5257	61.7
Liver disease	2671	4.7	985	8.2	830	9.1	681	8.0
Cancer	4369	7.7	1080	9.0	780	8.5	1029	12.1
Patient-reported medications								
Statins	14,933	26.2	3631	30.3	2975	32.6	2733	32.1
ACEs/ARBs	11,517	20.2	2695	22.5	2256	24.7	1879	22.0
NSAIDS	5908	10.4	1092	9.1	873	9.6	618	7.2
Steroids	6827	12.0	1556	13.0	1144	12.5	882	10.3
PPIs	9966	17.5	2269	19.0	1878	20.6	1749	20.5

*COPD* chronic obstructive pulmonary disease, *ACE* angiotensin-converting enzyme, *ARB* angiotensin II receptor blocker, *NSAIDS* non-steroidal anti-inflammatory drugs, *PPIs* proton-pump inhibitors, *SARS-CoV-2* severe acute respiratory syndrome coronavirus 2

*Includes acute and chronic kidney disease

The most common comorbidities among the patients hospitalized with COVID-19 overall were hypertension (40.8%), kidney disease (31.0%), diabetes (29.5%), and obesity (23.8%) (Table 1). In general, the prevalence of these comorbidities was higher among patients who experienced one of the outcomes of interest, compared to the broader hospitalized population. Among patients who received critical care, 48.9% had hypertension, 51.0% had kidney disease, 40.5% had diabetes, and 32.3% were obese. Among patients who received mechanical ventilation, 49.2% had hypertension, 55.9% had kidney disease, 42.9% had diabetes, and 34.8% were obese. Among patients who died, 47.3% had hypertension, 61.7% had kidney disease, and 39.6% had diabetes; 22.2% were obese, slightly less than hospitalized patients overall. Statins and angiotensin-converting enzyme inhibitors (ACEs)/angiotensin receptor blockers (ARBs) were the most prevalent patient-reported medications (26.2% and 20.2%, respectively, overall).

Table 2 presents the distributions of vital signs, laboratory values, symptoms, diagnoses, and treatments received during hospitalization among COVID-19 patients. The median duration of hospitalization overall was 6 days (IQR: 4, 11). The duration was longer among patients that experienced an outcome: 11 days (IQR: 6, 19) for critical care, 15 days (IQR: 8, 24) for mechanical ventilation, and 10 days (IQR: 5, 17) for death. Among hospitalized patients overall, 6.6% had a temperature > 38 degrees Celsius and 10.1% had an oxygen saturation < 90%. Markers of inflammation and coagulation were elevated for many patients, including 85.7% of patients with C-reactive protein (CRP) > 10 mg/L and 76.8% of patients with D-dimer > 250 ng/mL (DDU).

**Table 2:** Laboratory results, symptoms, diagnoses, and interventions during hospitalization among hospitalized COVID-19 patients, overall and by outcome

	Overall		Critical Care		Mechanical ventilation		Death	
	N	%	N	%	N	%	N	%
Duration of Hospitalization (median, IQR)	6.0	(4, 11)	11.0	(6, 19)	15.0	(8, 24)	10.0	(5, 17)
Observations (median, IQR)								
Temperature, °C	36.8	(36.5, 37.1)	36.8	(36.5, 37.2)	36.8	(36.5, 37.3)	36.8	(36.4, 37.4)
> 38	3,609	6.6	971	8.3	929	10.4	981	11.9
Oxygen saturation (SpO_2_), %	95.0	(93.0, 97.3)	95.0	(92.0, 97.2)	95.0	(92.0, 97.4)	95.0	(91.0, 97.0)
< 90	2457	10.1	1193	14.9	1107	14.7	1051	17.6
Platelet count × 10^9^ per L	224.0	(169.0, 298.0)	228.0	(165.0, 309.0)	225.0	(161.0, 306.0)	200.0	(143.0, 271.0)
< 150	9355	17.0	2324	19.7	1,879	20.8	2334	28.1
C-reactive protein, mg/L	60.5	(21.1, 124.0)	86.3	(34.1, 163.0)	97.7	(40.3, 178.0)	107.9	(51.0, 181.3)
> 10	30,979	85.7	8353	90.5	6469	92.1	5773	94.1
Ferritin, ng/mL	478.0	(219.1, 942.0)	632.0	(309.0, 1175.5)	673.1	(338.0, 1284.7)	689.0	(339.1, 1309.9)
> 300	22,216	66.3	6371	75.6	5141	78.0	4394	78.3
Lactate dehydrogenase, U/L	310.0	(232.0, 428.0)	377.0	(277.0, 526.0)	406.0	(294.0, 566.0)	405.0	(285.0, 571.0)
> 280	19,726	58.9	6235	73.9	5266	78.4	4384	75.8
D-Dimer, ng/mL	465.0	(264.0, 880.0)	680.0	(376.5, 1345.0)	773.8	(429.5, 1616.0)	855.0	(480.0, 1695.0)
> 250	19,021	76.8	5024	87.2	4006	89.7	3587	92.5
Fibrinogen, mg/dL	529.0	(403.0, 667.0)	546.0	(400.0, 700.0)	557.0	(403.0, 700.0)	537.0	(394.0, 693.0)
> 400	12,116	75.5	3750	74.9	3309	75.2	2501	74.2
Symptoms								
Hypoxemia	14,718	25.8	3968	33.2	3231	35.4	2719	31.9
Fever	9158	16.1	2617	21.9	2035	22.3	1641	19.2
Cough	7182	12.6	1656	13.8	1266	13.9	1032	12.1
Nausea/Vomiting	3711	6.5	814	6.8	547	6.0	345	4.0
Malaise and fatigue	7483	13.1	2242	18.7	1723	18.9	1484	17.4
Dyspnea or shortness of breath	15,979	28.0	4472	37.4	3781	41.4	2971	34.8
Diagnoses								
Acute respiratory failure	25,525	44.8	9472	79.2	7825	85.7	6187	72.6
Pneumonia	33,946	59.6	9771	81.6	7738	84.7	6565	77.0
Sepsis	12,646	22.2	5812	48.6	4995	54.7	4168	48.9
Coagulation defects or hemorrhagic conditions	3490	6.1	1715	14.3	1456	15.9	1101	12.9
Arrhythmia	4799	8.4	2112	17.6	1772	19.4	1439	16.9
Heart failure	7934	13.9	2775	23.2	2380	26.1	2441	28.6
MI	3437	6.0	1535	12.8	1293	14.2	1251	14.7
Kidney disease*	17,694	31.0	6103	51.0	5110	55.9	5257	61.7
Liver disease	2671	4.7	985	8.2	830	9.1	681	8.0
Treatments								
Chloroquine/Hydroxychloroquine	8852	15.5	2805	23.4	2632	28.8	2084	24.4
Lopinavir/Ritonavir	427	0.7	205	1.7	181	2.0	166	1.9
Remdesivir	12,990	22.8	3274	27.4	2829	31.0	1901	22.3
Dexamethasone	16,698	29.3	3968	33.2	3582	39.2	2520	29.6
ACEs/ARBs	12,313	21.6	2492	20.8	2112	23.1	1546	18.1
Anticoagulants	45,509	79.8	10,569	88.3	8286	90.7	7362	86.3
Immunosuppressants	3009	5.3	1409	11.8	1344	14.7	799	9.4
Antibacterials for systemic use	38,050	66.8	9373	78.3	7927	86.8	6967	81.7
Antivirals for systemic use	1847	3.2	633	5.3	555	6.1	434	5.1
Corticosteroids for systemic use	26,615	46.7	6897	57.6	6320	69.2	4699	55.1

*MI* myocardial infarction, *ACE* angiotensin-converting enzyme, *ARB* angiotensin II receptor blocker, *ICU* intensive care unit

*Includes acute and chronic kidney disease

The most common symptoms during hospitalization among COVID-19 patients were dyspnea (28.0%) and hypoxemia (25.8%) (Table 2). Prevalence of these symptoms was higher among patients who received critical care (37.4% and 33.2%, respectively), those who received ventilation (41.4% and 35.4%, respectively), and those who died (34.8% and 31.9%, respectively). Relatedly, the most prevalent diagnoses during hospitalization among patients overall were pneumonia (59.6%) and acute respiratory failure (44.8%). These diagnoses were even more common among those with an outcome, with the highest prevalence observed among patients that received mechanical ventilation (acute respiratory failure, 85.7%; pneumonia, 84.7%). Anticoagulants were the most prevalent treatment, received by 79.8% of hospitalized patients with COVID-19 overall during their hospitalization. Among other treatments, 15.5% of patients received chloroquine or hydroxychloroquine, 29.3% received dexamethasone, and 22.8% received remdesivir.

### Associations between covariates and serious outcomes

#### Baseline demographics and comorbidities

**Fig. 2:**
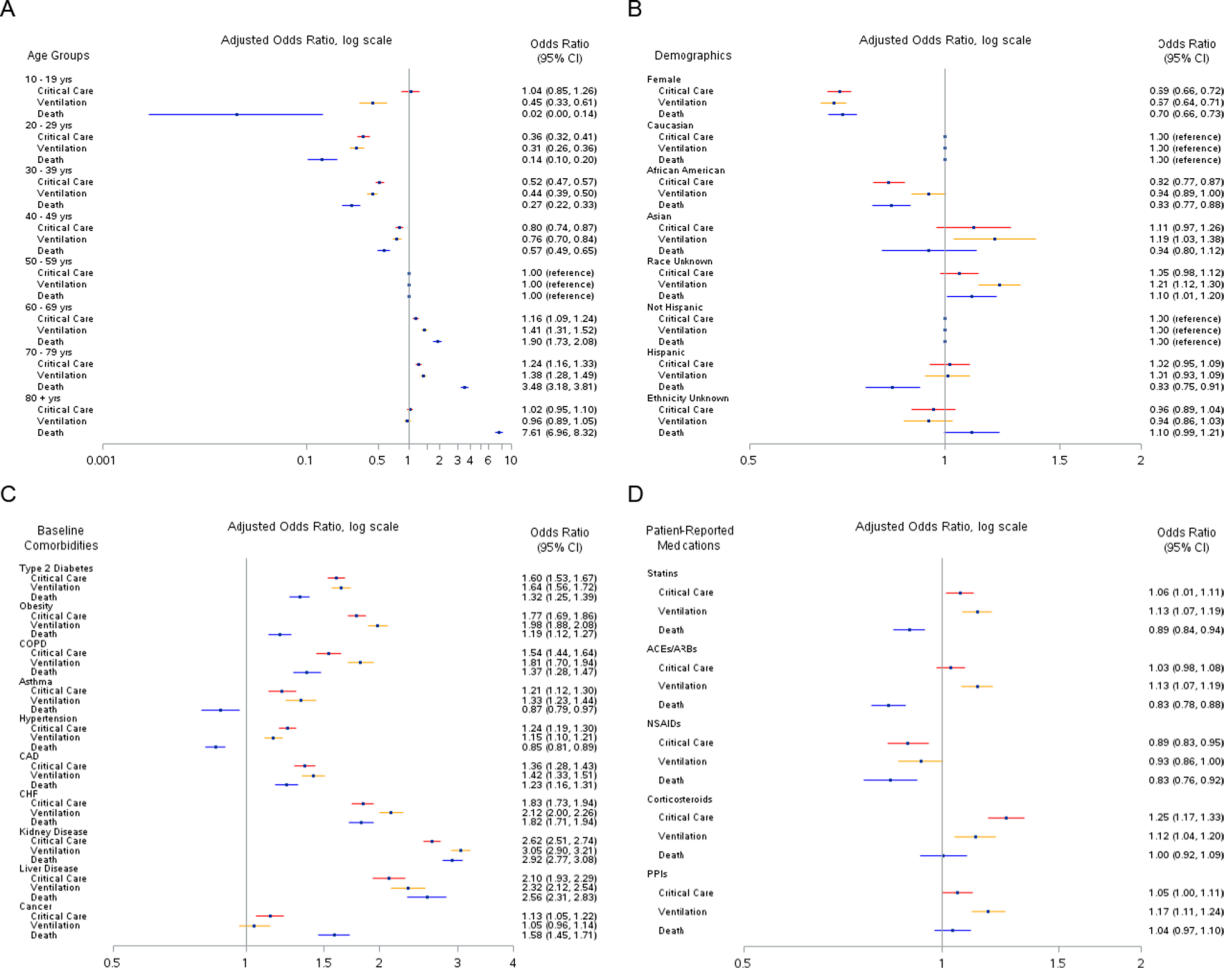
Associations between Baseline Characteristics and Critical Care, Mechanical Ventilation, and Death among Hospitalized COVID-19 Patients.** A** Age groups;** B** Demographics;** C** Baseline comorbidities;** D** Patient-reported medications. COPD, chronic obstructive pulmonary disease; CAD, coronary artery disease; CHF, congestive heart failure; ACE, angiotensin-converting enzyme; ARB, angiotensin II receptor blocker; NSAIDS, non-steroidal anti-inflammatory drugs; PPIs, proton-pump inhibitors. Logistic regression models were used to estimate odds ratios (OR) and corresponding 95% confidence intervals (CI) adjusted for age, gender, region, race, and week of cohort entry

Figure 2 presents the associations between baseline characteristics and receipt of critical care, ventilation, and death adjusted for age, gender, region, race, and week of cohort entry. Unadjusted ORs for the associations between covariates and outcomes are provided in Additional file 1: Tables S1–S3. Age was associated with all 3 outcomes, particularly death; the OR for patients ≥ 80 years of age compared to those 50–59 years of age was 7.61 (95% CI, 6.96–8.32) (Fig. 2A). Female patients were less likely to experience any of the 3 outcomes compared to males. Regarding race, hospitalized patients that received critical care, ventilation, or died were less likely to be African American compared to Caucasian (critical care: OR, 0.82; 95% CI, 0.77–0.87; mechanical ventilation: OR, 0.94; 95% CI, 0.89–1.00; death: OR, 0.83; 95% CI, 0.77–0.88) (Fig. 2B).

After adjusting for demographic variables, several comorbidities at baseline were associated with serious outcomes (Fig. 2C). Comorbidities associated with higher odds of all 3 outcomes included diabetes, obesity, chronic obstructive pulmonary disease, coronary artery disease, congestive heart failure, kidney disease, and liver disease. Asthma, hypertension, and cancer showed varying associations with each outcome. In the unadjusted model, hypertension was positively associated with death (OR, 1.37; 95% CI, 1.30–1.43; Additional file 1: Table S3). However, once adjusted for demographic variables, there was an inverse association between hypertension and death (OR, 0.85; 95% CI, 0.81–0.89). Asthma was associated with higher odds of critical care (OR, 1.21; 95% CI, 1.12–1.30) and ventilation (OR, 1.33; 95% CI, 1.23–1.44), but lower odds of death (OR, 0.87; 95% CI, 0.79–0.97).

Among select patient-reported medications at baseline, use of statins, corticosteroids, and PPIs was positively associated with receipt of critical care and ventilation, but not death (Fig. 2D). Statins, ACEs/ARBs, and NSAIDs showed inverse associations with death.

#### Vital signs and laboratory values

**Fig. 3:**
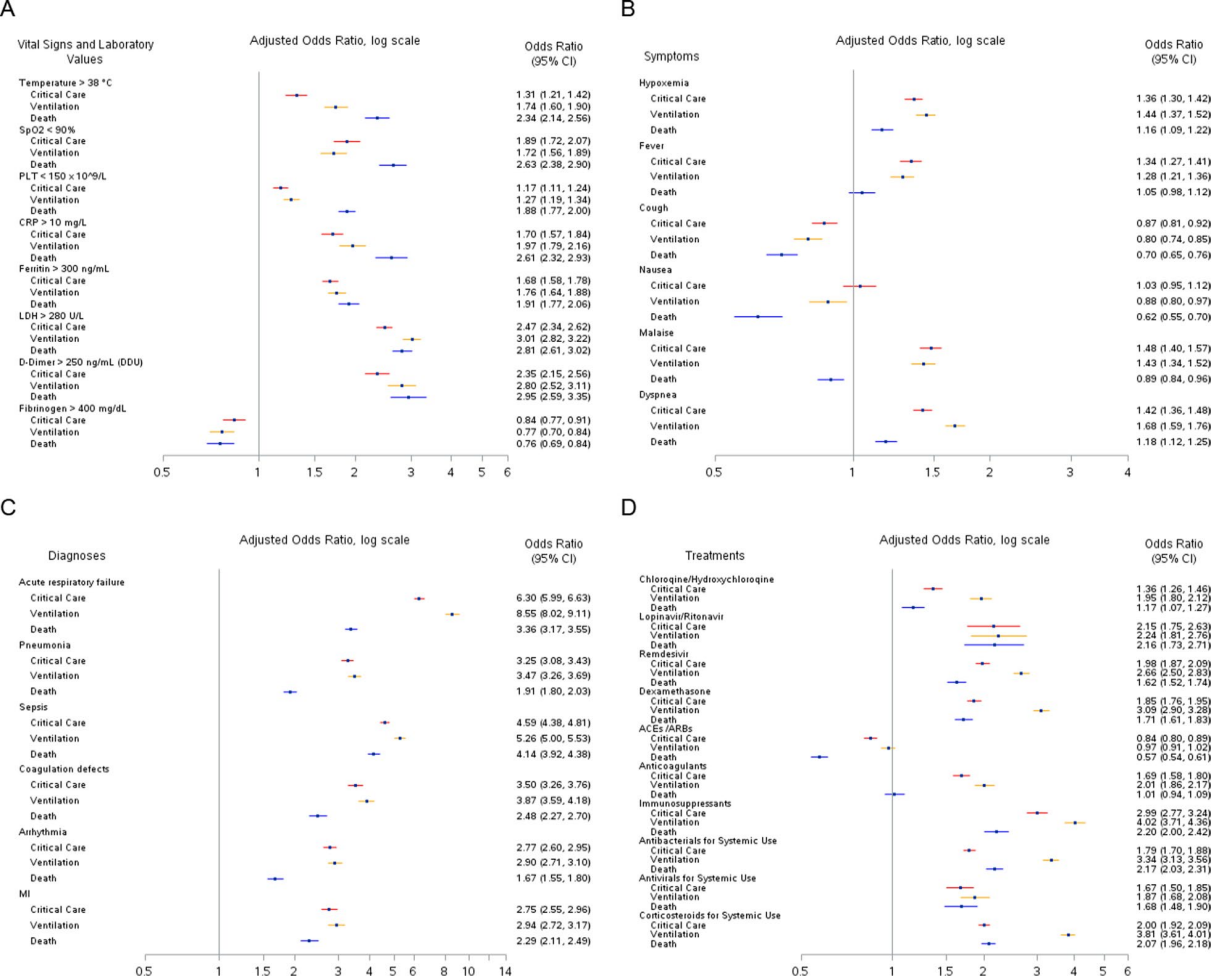
Associations between Clinical Variables During Hospitalization and Critical Care, Mechanical Ventilation, and Death among Hospitalized COVID-19 Patients.** A** Vital signs and laboratory values;** B** Symptoms;** C** Diagnoses;** D** Treatments. SpO2, oxygen saturation; PLT, platelet count; CRP, C-reactive protein; LDH, lactate dehydrogenase; MI, myocardial infarction; ACE, angiotensin-converting enzyme inhibitors; ARB, angiotensin II receptor blocker Logistic regression models were used to estimate odds ratios (OR) and corresponding 95% confidence intervals (CI) adjusted for age, gender, region, race, and week of cohort entry

Figure 3 presents the adjusted ORs for the associations between clinical characteristics during hospitalization and receipt of critical care, ventilation, and death among hospitalized patients with COVID-19. Measurements of temperature, CRP, ferritin, lactate dehydrogenase (LDH), and D-dimer that exceeded the clinically-relevant cutpoint were significantly associated with an increased risk of all 3 serious outcomes in adjusted models (Fig. 3A). Likewise, measurements of oxygen saturation and platelets that were less than the clinically-relevant cutpoint were also significantly associated with an increased risk of all 3 serious outcomes. D-dimer and LDH measured during hospitalization had the highest adjusted ORs for the association with death: OR, 2.95 (95% CI, 2.59–3.35) for D-dimer > 250 ng/mL (DDU) and OR, 2.81 (95% CI, 2.61–3.02) for LDH > 280 U/L. Fibrinogen > 400 mg/dL was associated with a lower risk of all 3 outcomes.

**Fig. 4:**
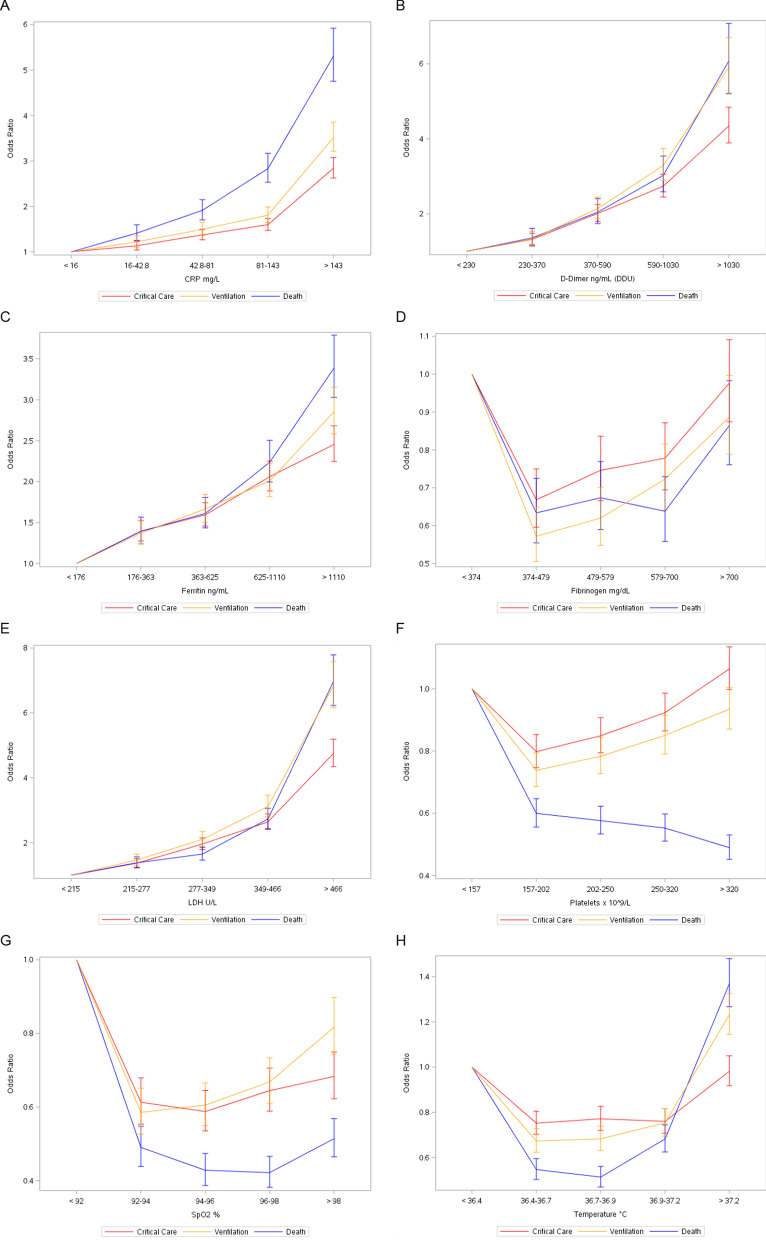
Associations between Vital Signs and Laboratory Markers During Hospitalization and Critical Care, Mechanical Ventilation, and Death among Hospitalized COVID-19 Patients.** A** C-reactive protein;** B** D-dimer;** C** Ferritin;** D** Fibrinogen;** E** Lactate dehydrogenase;** F** Platelets;** G** Oxygen saturation;** H** Temperature. SpO2, oxygen saturation; CRP, C-reactive protein; LDH, lactate dehydrogenase; Logistic regression models were used to estimate odds ratios (OR) and corresponding 95% confidence intervals (CI) adjusted for age, gender, region, race, and week of cohort entry

The associations between each biomarker in quintiles and serious outcomes are shown in Fig. 4. For CRP, D-dimer, ferritin, and LDH, the relationships appeared linear for all 3 outcomes (p values for linear trend < 0.0001, Additional file 1: Tables S1–S3). For temperature, oxygen saturation, and platelets, the relationships were less linear, although many of the p values for linear trend were < 0.0001. For fibrinogen, the associations with all 3 outcomes were non-linear, with p values of 0.44 for critical care, 0.92 for mechanical ventilation, and 0.09 for death. The biomarkers that showed the strongest associations with outcomes were LDH and D-dimer. The ORs for the 5th (> 466 U/L) versus 1st (< 215 U/L) quintiles of LDH were 4.75 (95% CI, 4.35–5.19) for critical care, 6.84 (95% CI, 6.16–7.58) for ventilation, and 6.97 (95% CI, 6.23–7.79) for death. Likewise, for D-dimer, the ORs for the 5th (> 1030 ng/mL) versus 1st quintile (< 230 ng/mL) were 4.34 (95% CI, 3.89–4.84) for critical care, 5.90 (95% CI, 5.19–6.71) for ventilation, and 6.07 (95% CI, 5.21–7.08) for death (Additional file 1: Tables S1–S3).

#### Symptoms and diagnoses during hospitalization

Dyspnea and hypoxemia were positively associated with all 3 serious outcomes in adjusted models, with both showing the strongest association with receipt of ventilation (dyspnea: OR, 1.68; 95% CI, 1.59–1.76; hypoxemia: OR, 1.44; 95% CI, 1.37–1.52) (Fig. 3B). In contrast, cough was associated with lower odds of all 3 outcomes (critical care: OR, 0.87; 95% CI, 0.81–0.92; mechanical ventilation: OR, 0.80; 95% CI, 0.74–0.85; death: OR, 0.70; 95% CI, 0.65–0.76). Patients with nausea and vomiting as well as malaise also had lower odds of death.

All selected in-hospital diagnoses showed positive associations with each of the 3 serious outcomes (Fig. 3C). The strongest associations were observed for acute respiratory failure (critical care: OR, 6.30; 95% CI, 5.99–6.63; ventilation: OR, 8.55; 95% CI, 8.02–9.11; death: OR, 3.36; 95% CI, 3.17–3.55) and sepsis (critical care: OR, 4.59; 95% CI, 4.38–4.81; ventilation: OR, 5.26; 95% CI, 5.00–5.53; death: OR, 4.14; 95% CI, 3.92–4.38).

#### Treatments received during hospitalization

With the exception ACEs/ARBs, all of the selected treatments were associated with higher odds of serious outcomes (Fig. 3D). The highest ORs were observed for the associations between treatments and receipt of ventilation, ranging from 1.87 (95% CI, 1.68–2.08) for antivirals to 4.02 (95% CI, 3.71–4.36) for immunosuppressants. Treatment with ACEs/ARBs was inversely associated with receipt of critical care (OR, 0.84; 95% CI, 0.80–0.89) and death (OR, 0.57; 95% CI, 0.54–0.61).

## Discussion

In this study, we identified and described patients who experienced a serious outcome (critical care, mechanical ventilation, and death) among 56,996 hospitalized patients with COVID-19 within a large, EHR database. We conducted an evaluation of the association of many demographic and clinical characteristics with these outcomes in order to identify potential signals for experiencing a serious outcome. As was observed in prior studies [7–10], older age and male gender were associated with higher risk of serious outcomes, along with comorbidities such as diabetes and kidney disease. We also observed associations with clinical characteristics measured during hospitalization, including several laboratory markers, symptoms, and diagnoses.

In this study of hospitalized patients with COVID-19, we found that African-Americans and women were at lower risk of experiencing a serious outcome compared to Caucasians and men, respectively. Since the start of the pandemic, African-Americans have been more likely than Caucasians to contract and be hospitalized with COVID-19 [11]. Nonetheless, evidence suggests that once hospitalized, they do not have a higher risk of adverse outcomes [10, 12–14]. In contrast, there does not appear to be sex difference in number of confirmed cases of COVID-19, but the death rate has been higher in men than women [15]. The reason for the sex difference in rate of mortality among patients with COVID-19 remains unknown, but it has been suggested that the mechanism involves a combination of biological and psychosocial factors [16].

Hypertension was the most prevalent comorbidity among hospitalized COVID-19 patients in this study and its prevalence was even higher among patients with a serious outcome. Hypertension was positively associated with receipt of critical care and mechanical ventilation, but inversely associated with mortality after adjusting for age and other demographics. Findings in this study are consistent with previous studies that have found hypertension to be common among adults diagnosed with COVID-19, but not associated with mortality after adjusting for covariates [14, 17]. Thus, while there may be overrepresentation of hypertension among adults with COVID-19, it appears this association may be confounded by age and other covariates, and possibly affected by treatment.

In the current study, treatment with ACEs/ARBs during hospitalization was associated with lower odds of critical care and mortality. It is recommended that patients who are prescribed ACEs/ARBs for cardiovascular disease continue taking these medications if hospitalized with COVID-19 [18, 19]. With the exception of ACEs/ARBs, we observed that many treatments received during hospitalization were associated with higher odds of receipt of critical care, mechanical ventilation, or death. An explanation for this finding may be that most medications, particularly those that were investigational, were only recommended for use among patients with severe disease. For example, remdesivir and dexamethasone are recommended for hospitalized COVID-19 patients that require supplemental oxygen [19]. As of 15 December 2021, remdesivir is the only FDA-approved drug for the treatment of COVID-19, although emergency use authorizations have been issued for multiple anti-SARS-CoV-2 monoclonal antibody products (i.e., bamlanivimab plus etesevimab, casirivimab plus imdevimab, and sotrovimab) [19]. Other medications are under investigation.

Several vital signs and laboratory results were associated with serious outcomes in COVID-19 patients in this study, with the strongest associations observed for LDH, D-dimer, and CRP. LDH is an enzyme found within cells in almost all organ systems [20]. Its levels rise when the body’s tissues are damaged. Recent studies have found that high levels of LDH may be predictive of COVID-19 severity and death [17, 20]. Similarly, increased D-dimer, an indicator of coagulopathy, has been linked to higher risk of mortality in COVID-19 patients [8, 21]. CRP is an inflammatory marker and has been shown to be elevated in patients with severe COVID-19 disease [9, 12, 22]. The findings in the current study are consistent with these smaller studies and, taken together, suggest these laboratory markers, if measured soon after admission, may help clinicians triage patients who may be at higher risk of progression to severe disease.

The current study was based on an analysis of EHR data, which are valuable for the examination of clinical health outcomes and treatment patterns. Nonetheless, all EHR databases have inherent limitations because the data are collected for the purpose of clinical patient management, not research. Unlike in clinical trials, where the collection of clinical and laboratory measures is standardized, the Optum EHR includes real-world clinical data obtained from multiple medical and laboratory settings used for patient care. Because data are not collected in a systematic way, clinical measurements (e.g., vital signs and laboratory results) were not available for all patients.

Additionally, the presence of a diagnosis code in EHR data may not represent the actual presence of disease, as the diagnosis code may be incorrectly coded, or included as rule-out criteria rather than actual disease. We assumed the absence of a diagnosis code meant the patient did not have the disease. This assumption may be a reason why we observed an inverse association between cough and serious outcomes; in a patient who is very ill, severe symptoms like dyspnea and hypoxemia may be more likely to be recorded than minor symptoms such as cough. Furthermore, it is possible that some comorbidities and medications may not have been captured as health care encounters with medical providers who do not contract with Optum would not be observed.

The median duration of hospitalization among patients who died was 10 days. However, deaths were identified within all data available, not only during hospitalization. As such, it is possible that duration of hospitalization may have been shorter among patients that died during hospitalization. An additional limitation of the EHR database is the data lag at the time of data extraction, which likely resulted in an underestimation of the number of deaths. Finally, residual confounding is a concern as we only adjusted for demographic covariates.

## Conclusion

In summary, we utilized an exploratory, data-driven approach to identify many clinical characteristics that were associated with receipt of critical care, mechanical ventilation, and death among patients hospitalized with COVID-19. As more continues to be learned about COVID-19 by clinicians and researchers, future studies should move toward causal inference and focus on identifying the etiologic factors and mechanisms responsible for some patients experiencing more severe COVID-19 disease.

## Supplementary Information

**Additional file 1.** Supplemental tables.

## Data Availability

The de-identified database used for the current study is not publicly available, but is available from Optum through a data license agreement. More information can be found at the following website: https://www.optum.com/business/solutions/life-sciences/real-world-data/ehr-data.html.

## Abbreviations

ACE: Angiotensin-converting enzyme inhibitor
ARB: Angiotensin receptor blockers
ATC: Anatomical therapeutic chemical
CI: Confidence interval
COVID-19: Coronavirus disease 2019
CPT-4: Current Procedural Terminology, 4th Edition
CRP: C-reactive protein
ECMO: Extracorporeal membrane oxygenation
EHR: Electronic health record
EMR: Electronic medical record
ICD-10-CM: International Classification of Diseases, 10th Revision, Clinical Modification
ICD-10-PCS: International Classification of Diseases, 10th Revision, Procedural Coding System
IQR: Interquartile range
LDH: Lactate dehydrogenase
NSAID: Nonsteroidal anti-inflammatory drug
OR: Odds ratio
SARS-CoV-2: Severe acute respiratory syndrome coronavirus 2
US: United States
