# Supplementary material for: Predictors of critical care, mechanical ventilation, and mortality among hospitalized patients with COVID-19 in an electronic health record database
Source: BMC Infect Dis. 2022 Apr 29;22:413. doi: 10.1186/s12879-022-07383-6 (PMC9051491; doi:10.1186/s12879-022-07383-6)
Supplement: Supplementary file 1 — Additional file 1. Supplemental tables. [file 12879_2022_7383_MOESM1_ESM.docx]

| **Supplemental Table 1. Association of Baseline Characteristics and Clinical Variables During Hospitalization with Critical Care among Hospitalized COVID-19 Patients** | | | | | | | | | | | | |  |  |  |
| --- | --- | --- | --- | --- | --- | --- | --- | --- | --- | --- | --- | --- | --- | --- | --- |
|  | | **Unadjusted Model** | | | | | **Adjusted Model*** | | | | | | |  |  |
|  |  | **OR** | | **Lower Bound** | **Upper Bound** | | **OR** | | **Lower Bound** | | **Upper Bound** | | |  |  |
| *Baseline* | |  | |  |  | |  | |  | |  | | |  |  |
| **Age** | |  | |  |  | |  | |  | |  | | |  |  |
| <10 | | 0.90 | | 0.71 | 1.16 | | 0.90 | | 0.70 | | 1.16 | | |  |  |
| 10-19 | | 0.87 | | 0.72 | 1.06 | | 1.04 | | 0.85 | | 1.26 | | |  |  |
| 20-29 | | 0.31 | | 0.27 | 0.35 | | 0.36 | | 0.32 | | 0.41 | | |  |  |
| 30-39 | | 0.47 | | 0.43 | 0.52 | | 0.52 | | 0.47 | | 0.57 | | |  |  |
| 40-49 | | 0.81 | | 0.74 | 0.88 | | 0.80 | | 0.74 | | 0.87 | | |  |  |
| 50-59 | | 1.00 | | Reference | | | 1.00 | | Reference | | | | |  |  |
| 60-69 | | 1.13 | | 1.06 | 1.21 | | 1.16 | | 1.09 | | 1.24 | | |  |  |
| 70-79 | | 1.17 | | 1.09 | 1.25 | | 1.24 | | 1.16 | | 1.33 | | |  |  |
| 80+ | | 0.95 | | 0.89 | 1.02 | | 1.02 | | 0.95 | | 1.10 | | |  |  |
| **Gender** | |  | |  |  | |  | |  | |  | | |  |  |
| Female | | 0.62 | | 0.60 | 0.65 | | 0.69 | | 0.66 | | 0.72 | | |  |  |
| Male | | 1.00 | | Reference | | | 1.00 | | Reference | | | | |  |  |
| **Race** | |  | |  |  | |  | |  | |  | | |  |  |
| African American | | 0.90 | | 0.85 | 0.95 | | 0.82 | | 0.77 | | 0.87 | | |  |  |
| Asian | | 1.21 | | 1.07 | 1.37 | | 1.11 | | 0.97 | | 1.26 | | |  |  |
| Caucasian | | 1.00 | | Reference | | | 1.00 | | Reference | | | | |  |  |
| Other/Unknown | | 1.01 | | 0.95 | 1.06 | | 1.05 | | 0.98 | | 1.12 | | |  |  |
| **Ethnicity** | |  | |  |  | |  | |  | |  | | |  |  |
| Hispanic | | 0.94 | | 0.89 | 0.99 | | 1.02 | | 0.95 | | 1.09 | | |  |  |
| Not Hispanic | | 1.00 | | Reference | | | 1.00 | | Reference | | | | |  |  |
| Unknown | | 0.99 | | 0.92 | 1.06 | | 0.96 | | 0.89 | | 1.04 | | |  |  |
| **Comorbidities** | |  | |  |  | |  | |  | |  | | |  |  |
| Diabetes | | 1.88 | | 1.80 | 1.96 | | 1.60 | | 1.53 | | 1.67 | | |  |  |
| Obesity | | 1.74 | | 1.66 | 1.82 | | 1.77 | | 1.69 | | 1.86 | | |  |  |
| Pulmonary disease | |  | |  |  | |  | |  | |  | | |  |  |
| COPD | | 1.82 | | 1.72 | 1.93 | | 1.54 | | 1.44 | | 1.64 | | |  |  |
| Asthma | | 1.11 | | 1.04 | 1.19 | | 1.21 | | 1.12 | | 1.30 | | |  |  |
| Cardiovascular disease | |  | |  |  | |  | |  | |  | | |  |  |
| Hypertension | | 1.52 | | 1.46 | 1.59 | | 1.24 | | 1.19 | | 1.30 | | |  |  |
| Coronary artery disease | | 1.72 | | 1.63 | 1.81 | | 1.36 | | 1.28 | | 1.43 | | |  |  |
| Congestive heart failure | | 2.12 | | 2.01 | 2.24 | | 1.83 | | 1.73 | | 1.94 | | |  |  |
| Kidney disease** | | 3.00 | | 2.88 | 3.13 | | 2.62 | | 2.51 | | 2.74 | | |  |  |
| Liver disease | | 2.31 | | 2.13 | 2.50 | | 2.10 | | 1.93 | | 2.29 | | |  |  |
| Cancer | | 1.26 | | 1.17 | 1.35 | | 1.13 | | 1.05 | | 1.22 | | |  |  |
| **Patient-reported medications** | |  | |  |  | |  | |  | |  | | |  |  |
| Statins | | 1.30 | | 1.24 | 1.36 | | 1.06 | | 1.01 | | 1.11 | | |  |  |
| ACEs/ARBs | | 1.19 | | 1.14 | 1.25 | | 1.03 | | 0.98 | | 1.08 | | |  |  |
| NSAIDS | | 0.84 | | 0.78 | 0.90 | | 0.89 | | 0.83 | | 0.95 | | |  |  |
| Steroids | | 1.13 | | 1.06 | 1.20 | | 1.25 | | 1.17 | | 1.33 | | |  |  |
| PPIs | | 1.13 | | 1.08 | 1.20 | | 1.05 | | 1.00 | | 1.11 | | |  |  |
| *During Hospitalization* | |  | |  |  | |  | |  | |  | | |  |  |
| **Observations (median, IQR)** | |  | |  |  | |  | |  | |  | | |  |  |
| Temperature, °C | |  | |  |  | |  | |  | |  | | |  |  |
| > 38 | | 1.39 | | 1.29 | 1.50 | | 1.31 | | 1.21 | | 1.42 | | |  |  |
| Quintiles | |  | |  |  | |  | |  | |  | | |  |  |
| < 36.4 | | 1.00 | | Reference | | | 1.00 | | Reference | | | | |  |  |
| 36.4 – 36.7 | | 0.76 | | 0.71 | 0.81 | | 0.75 | | 0.70 | | 0.80 | | |  |  |
| 36.7 – 36.9 | | 0.77 | | 0.72 | 0.82 | | 0.77 | | 0.72 | | 0.83 | | |  |  |
| 36.9 – 37.2 | | 0.76 | | 0.71 | 0.82 | | 0.76 | | 0.71 | | 0.82 | | |  |  |
| > 37.2 | | 1.04 | | 0.97 | 1.11 | | 0.98 | | 0.92 | | 1.05 | | |  |  |
| *P value for linear trend* | | <.0001 | |  |  | | 0.002 | |  | |  | | |  |  |
| Oxygen saturation (SpO2), % | |  | |  |  | |  | |  | |  | | |  |  |
| < 90 | | 2.07 | | 1.90 | 2.25 | | 1.89 | | 1.72 | | 2.07 | | |  |  |
| Quintiles | |  | |  |  | |  | |  | |  | | |  |  |
| < 92 | | 1.00 | | Reference | | | 1.00 | | Reference | | | | |  |  |
| 92 – 94 | | 0.56 | | 0.51 | 0.62 | | 0.61 | | 0.55 | | 0.68 | | |  |  |
| 94 – 96 | | 0.56 | | 0.51 | 0.60 | | 0.59 | | 0.54 | | 0.65 | | |  |  |
| 96 – 98 | | 0.68 | | 0.62 | 0.73 | | 0.64 | | 0.59 | | 0.71 | | |  |  |
| > 98 | | 0.55 | | 0.51 | 0.60 | | 0.68 | | 0.62 | | 0.75 | | |  |  |
| *P value for linear trend* | | <.0001 | |  |  | | <.0001 | |  | |  | | |  |  |
| Platelet count x 10^9^ per L | |  | |  |  | |  | |  | |  | | |  |  |
| < 150 | | 1.26 | | 1.19 | 1.33 | | 1.17 | | 1.11 | | 1.24 | | |  |  |
| Quintiles | |  | |  |  | |  | |  | |  | | |  |  |
| < 157 | | 1.00 | | Reference | | | 1.00 | | Reference | | | | |  |  |
| 157 - 202 | | 0.75 | | 0.70 | 0.80 | | 0.80 | | 0.75 | | 0.85 | | |  |  |
| 202 – 250 | | 0.77 | | 0.73 | 0.83 | | 0.85 | | 0.79 | | 0.91 | | |  |  |
| 250 – 320 | | 0.85 | | 0.80 | 0.91 | | 0.92 | | 0.86 | | 0.99 | | |  |  |
| > 320 | | 1.02 | | 0.96 | 1.09 | | 1.06 | | 1.00 | | 1.14 | | |  |  |
| *P value for linear trend* | | <.0001 | |  |  | | <.0001 | |  | |  | | |  |  |
| C-reactive protein, mg/L | |  | |  |  | |  | |  | |  | | |  |  |
| > 10 | | 1.79 | | 1.66 | 1.94 | | 1.70 | | 1.57 | | 1.84 | | |  |  |
| Quintiles | |  | |  |  | |  | |  | |  | | |  |  |
| < 16 | | 1.00 | | Reference | | | 1.00 | | Reference | | | | |  |  |
| 16 – 42.8 | | 1.15 | | 1.06 | 1.25 | | 1.13 | | 1.04 | | 1.23 | | |  |  |
| 42.8 – 81 | | 1.44 | | 1.32 | 1.56 | | 1.37 | | 1.26 | | 1.49 | | |  |  |
| 81 – 143 | | 1.70 | | 1.57 | 1.84 | | 1.60 | | 1.47 | | 1.73 | | |  |  |
| > 143 | | 3.06 | | 2.83 | 3.30 | | 2.84 | | 2.62 | | 3.07 | | |  |  |
| *P value for linear trend* | | <.0001 | |  |  | | <.0001 | |  | |  | | |  |  |
| Ferritin, ng/mL | |  | |  |  | |  | |  | |  | | |  |  |
| > 300 | | 1.81 | | 1.71 | 1.91 | | 1.68 | | 1.58 | | 1.78 | | |  |  |
| Quintiles | |  | |  |  | |  | |  | |  | | |  |  |
| <176 | | 1.00 | | Reference | | | 1.00 | | Reference | | | | |  |  |
| 176 – 363 | | 1.45 | | 1.32 | 1.58 | | 1.39 | | 1.28 | | 1.53 | | |  |  |
| 363 - 625 | | 1.67 | | 1.54 | 1.82 | | 1.59 | | 1.46 | | 1.74 | | |  |  |
| 625 – 1100 | | 2.19 | | 2.02 | 2.39 | | 2.06 | | 1.89 | | 2.25 | | |  |  |
| >1100 | | 2.67 | | 2.46 | 2.90 | | 2.45 | | 2.25 | | 2.68 | | |  |  |
| *P value for linear trend* | | <.0001 | |  |  | | <.0001 | |  | |  | | |  |  |
| Lactate dehydrogenase, U/L | |  | |  |  | |  | |  | |  | | |  |  |
| > 280 | | 2.43 | | 2.30 | 2.56 | | 2.47 | | 2.34 | | 2.62 | | |  |  |
| Quintiles | |  | |  |  | |  | |  | |  | | |  |  |
| < 215 | | 1.00 | | Reference | | | 1.00 | | Reference | | | | |  |  |
| 215 – 277 | | 1.35 | | 1.23 | 1.48 | | 1.37 | | 1.25 | | 1.51 | | |  |  |
| 277 - 349 | | 1.90 | | 1.74 | 2.08 | | 1.96 | | 1.79 | | 2.15 | | |  |  |
| 349 – 466 | | 2.52 | | 2.31 | 2.76 | | 2.64 | | 2.41 | | 2.89 | | |  |  |
| > 466 | | 4.50 | | 4.14 | 4.90 | | 4.75 | | 4.35 | | 5.19 | | |  |  |
| *P value for linear trend* | | <.0001 | |  |  | | <.0001 | |  | |  | | |  |  |
| D-Dimer, ng/mL | |  | |  |  | |  | |  | |  | | |  |  |
| > 250 | | 2.44 | | 2.25 | 2.66 | | 2.35 | | 2.15 | | 2.56 | | |  |  |
| Quintiles | |  | |  |  | |  | |  | |  | | |  |  |
| < 230 | | 1.00 | | Reference | | | 1.00 | | Reference | | | | |  |  |
| 230 - 370 | | 1.36 | | 1.21 | 1.52 | | 1.32 | | 1.18 | | 1.48 | | |  |  |
| 370 – 590 | | 2.01 | | 1.80 | 2.24 | | 2.01 | | 1.80 | | 2.25 | | |  |  |
| 590 – 1030 | | 2.76 | | 2.48 | 3.07 | | 2.74 | | 2.45 | | 3.05 | | |  |  |
| > 1030 | | 4.30 | | 3.88 | 4.77 | | 4.34 | | 3.89 | | 4.84 | | |  |  |
| *P value for linear trend* | | <.0001 | |  |  | | <.0001 | |  | |  | | |  |  |
| Fibrinogen, mg/dL | |  | |  |  | |  | |  | |  | | |  |  |
| > 400 | | 0.95 | | 0.88 | 1.03 | | 0.84 | | 0.77 | | 0.91 | | |  |  |
| Quintiles | |  | |  |  | |  | |  | |  | | |  |  |
| < 374 | | 1.00 | | Reference | | | 1.00 | | Reference | | | | |  |  |
| 374 – 479 | | 0.67 | | 0.60 | 0.75 | | 0.67 | | 0.60 | | 0.75 | | |  |  |
| 479 – 579 | | 0.79 | | 0.71 | 0.88 | | 0.75 | | 0.67 | | 0.84 | | |  |  |
| 579 - 700 | | 0.87 | | 0.78 | 0.97 | | 0.78 | | 0.69 | | 0.87 | | |  |  |
| > 700 | | 1.24 | | 1.12 | 1.37 | | 0.98 | | 0.87 | | 1.09 | | |  |  |
| *P value for linear trend* | | <.0001 | |  |  | | 0.44 | |  | |  | | |  |  |
| **Symptoms** | |  | |  |  | |  | |  | |  | | |  |  |
| Hypoxemia | | 1.58 | | 1.51 | 1.65 | | 1.36 | | 1.30 | | 1.42 | | |  |  |
| Fever | | 1.65 | | 1.57 | 1.73 | | 1.34 | | 1.27 | | 1.41 | | |  |  |
| Cough | | 1.15 | | 1.08 | 1.22 | | 0.87 | | 0.81 | | 0.92 | | |  |  |
| Nausea/Vomiting | | 1.06 | | 0.98 | 1.15 | | 1.03 | | 0.95 | | 1.12 | | |  |  |
| Malaise and fatigue | | 1.75 | | 1.66 | 1.85 | | 1.48 | | 1.40 | | 1.57 | | |  |  |
| Dyspnea or shortness of breath | | 1.74 | | 1.67 | 1.81 | | 1.42 | | 1.36 | | 1.48 | | |  |  |
| **Diagnoses** | |  | |  |  | |  | |  | |  | | |  |  |
| Acute respiratory failure | | 6.85 | | 6.53 | 7.19 | | 6.30 | | 5.99 | | 6.63 | | |  |  |
| Pneumonia | | 3.84 | | 3.65 | 4.03 | | 3.25 | | 3.08 | | 3.43 | | |  |  |
| Sepsis | | 5.28 | | 5.05 | 5.52 | | 4.59 | | 4.39 | | 4.81 | | |  |  |
| Coagulation defects or hemorrhagic conditions | | 4.08 | | 3.80 | 4.37 | | 3.50 | | 3.26 | | 3.76 | | |  |  |
| Arrhythmia | | 3.38 | | 3.18 | 3.59 | | 2.77 | | 2.60 | | 2.95 | | |  |  |
| Heart failure | | 2.33 | | 2.22 | 2.46 | | 2.03 | | 1.92 | | 2.15 | | |  |  |
| MI | | 3.34 | | 3.11 | 3.58 | | 2.75 | | 2.55 | | 2.96 | | |  |  |
| **Treatments** | |  | |  |  | |  | |  | |  | | |  |  |
| Chloroquine/Hydroxychloroquine | | 1.97 | | 1.88 | 2.08 | | 1.36 | | 1.26 | | 1.46 | | |  |  |
| lopinavir/Ritonavir | | 3.52 | | 2.91 | 4.26 | | 2.15 | | 1.75 | | 2.63 | | |  |  |
| Remdesivir | | 1.37 | | 1.31 | 1.43 | | 1.98 | | 1.87 | | 2.09 | | |  |  |
| Dexamethasone | | 1.26 | | 1.21 | 1.31 | | 1.85 | | 1.76 | | 1.95 | | |  |  |
| ACEs/ARBs | | 0.94 | | 0.90 | 0.99 | | 0.84 | | 0.80 | | 0.89 | | |  |  |
| Anticoagulants | | 2.18 | | 2.06 | 2.32 | | 1.69 | | 1.58 | | 1.80 | | |  |  |
| Immunosuppressants | | 3.62 | | 3.36 | 3.90 | | 2.99 | | 2.77 | | 3.24 | | |  |  |
| Antibacterials for systemic use | | 2.06 | | 1.96 | 2.16 | | 1.79 | | 1.70 | | 1.88 | | |  |  |
| Antivirals for systemic use | | 2.02 | | 1.83 | 2.22 | | 1.67 | | 1.50 | | 1.85 | | |  |  |
| Corticosteroids for systemic use | | 1.75 | | 1.68 | 1.82 | | 2.00 | | 1.92 | | 2.09 | | |  |  |
| Abbreviations: COPD, chronic obstructive pulmonary disease; ACE, angiotensin-converting enzyme; ARB, angiotensin II receptor blocker; NSAIDS, non-steroidal anti-inflammatory drugs; PPIs, proton-pump inhibitors | | | | | | | | | | | | | | |  |
| *Adjusted for age, gender, region, race, and week of cohort entry. | | | | | |  | |  | |  | |  | | | |
| **Includes acute and chronic kidney disease. |  | |  | | |  | |  | |  | |  | | | |

| **Supplemental Table 2. Association of Baseline Characteristics and Clinical Variables During Hospitalization with Intubation/Ventilation/ECMO among Hospitalized COVID-19 Patients** | | | | | | | | | | | | |  |  |
| --- | --- | --- | --- | --- | --- | --- | --- | --- | --- | --- | --- | --- | --- | --- |
|  | **Unadjusted Model** | | | | | | **Adjusted Model*** | | | | | | |  |
|  | **OR** | | **Lower Bound** | | **Upper Bound** | | **OR** | | | **Lower Bound** | **Upper Bound** | | |  |
| *Baseline* |  | |  | |  | |  | | |  |  | | |  |
| **Age** |  | |  | |  | |  | | |  |  | | |  |
| <10 | 0.39 | | 0.26 | | 0.57 | | 0.41 | | | 0.28 | 0.61 | | |  |
| 10-19 | 0.36 | | 0.27 | | 0.49 | | 0.45 | | | 0.33 | 0.61 | | |  |
| 20-29 | 0.25 | | 0.21 | | 0.29 | | 0.31 | | | 0.26 | 0.36 | | |  |
| 30-39 | 0.39 | | 0.35 | | 0.44 | | 0.44 | | | 0.39 | 0.50 | | |  |
| 40-49 | 0.77 | | 0.70 | | 0.84 | | 0.76 | | | 0.70 | 0.84 | | |  |
| 50-59 | 1.00 | | Reference | | | | 1.00 | | | Reference | | | |  |
| 60-69 | 1.34 | | 1.25 | | 1.44 | | 1.41 | | | 1.31 | 1.52 | | |  |
| 70-79 | 1.27 | | 1.18 | | 1.37 | | 1.38 | | | 1.28 | 1.49 | | |  |
| 80+ | 0.86 | | 0.79 | | 0.93 | | 0.96 | | | 0.89 | 1.05 | | |  |
| **Gender** |  | |  | |  | |  | | |  |  | | |  |
| Female | 0.59 | | 0.57 | | 0.62 | | 0.67 | | | 0.64 | 0.71 | | |  |
| Male | 1.00 | | Reference | | | | 1.00 | | | Reference | | | |  |
| **Race** |  | |  | |  | |  | | |  |  | | |  |
| African American | 1.03 | | 0.97 | | 1.09 | | 0.94 | | | 0.89 | 1.00 | | |  |
| Asian | 1.25 | | 1.08 | | 1.43 | | 1.19 | | | 1.03 | 1.38 | | |  |
| Caucasian | 1.00 | | Reference | | | | 1.00 | | | Reference | | | |  |
| Other/Unknown | 1.12 | | 1.05 | | 1.18 | | 1.21 | | | 1.12 | 1.30 | | |  |
| **Ethnicity** |  | |  | |  | |  | | |  |  | | |  |
| Hispanic | 0.92 | | 0.86 | | 0.98 | | 1.01 | | | 0.93 | 1.09 | | |  |
| Not Hispanic | 1.00 | | Reference | | | | 1.00 | | | Reference | | | |  |
| Unknown | 1.07 | | 0.99 | | 1.15 | | 0.94 | | | 0.86 | 1.03 | | |  |
| **Comorbidities** |  | |  | |  | |  | | |  |  | | |  |
| Diabetes | 2.03 | | 1.94 | | 2.13 | | 1.64 | | | 1.56 | 1.72 | | |  |
| Obesity | 1.94 | | 1.84 | | 2.03 | | 1.98 | | | 1.88 | 2.08 | | |  |
| Pulmonary disease |  | |  | |  | |  | | |  |  | | |  |
| COPD | 2.15 | | 2.02 | | 2.29 | | 1.81 | | | 1.70 | 1.94 | | |  |
| Asthma | 1.21 | | 1.12 | | 1.31 | | 1.33 | | | 1.23 | 1.44 | | |  |
| Cardiovascular disease |  | |  | |  | |  | | |  |  | | |  |
| Hypertension | 1.51 | | 1.44 | | 1.58 | | 1.15 | | | 1.10 | 1.21 | | |  |
| Coronary artery disease | 1.79 | | 1.69 | | 1.90 | | 1.42 | | | 1.33 | 1.51 | | |  |
| Congestive heart failure | 2.39 | | 2.26 | | 2.53 | | 2.12 | | | 2.00 | 2.26 | | |  |
| Kidney disease** | 3.56 | | 3.40 | | 3.73 | | 3.05 | | | 2.90 | 3.21 | | |  |
| Liver disease | 2.50 | | 2.29 | | 2.72 | | 2.32 | | | 2.12 | 2.54 | | |  |
| Cancer | 1.15 | | 1.06 | | 1.25 | | 1.05 | | | 0.96 | 1.14 | | |  |
| **Patient-reported medications** |  | |  | |  | |  | | |  |  | | |  |
| Statins | 1.45 | | 1.38 | | 1.52 | | 1.13 | | | 1.07 | 1.19 | | |  |
| ACEs/ARBs | 1.37 | | 1.30 | | 1.44 | | 1.13 | | | 1.07 | 1.19 | | |  |
| NSAIDS | 0.90 | | 0.83 | | 0.97 | | 0.93 | | | 0.86 | 1.00 | | |  |
| Steroids | 1.06 | | 0.99 | | 1.14 | | 1.12 | | | 1.04 | 1.20 | | |  |
| PPIs | 1.27 | | 1.20 | | 1.35 | | 1.17 | | | 1.11 | 1.24 | | |  |
| *During Hospitalization* |  | |  | |  | |  | | |  |  | | |  |
| **Observations (median, IQR)** |  | |  | |  | |  | | |  |  | | |  |
| Temperature, °C |  | |  | |  | |  | | |  |  | | |  |
| > 38 | 1.88 | | 1.74 | | 2.03 | | 1.74 | | | 1.60 | 1.90 | | |  |
| Quintiles |  | |  | |  | |  | | |  |  | | |  |
| < 36.4 | 1.00 | | Reference | | | | 1.00 | | | Reference | | | |  |
| 36.4 – 36.7 | 0.68 | | 0.63 | | 0.73 | | 0.67 | | | 0.62 | 0.73 | | |  |
| 36.7 – 36.9 | 0.67 | | 0.62 | | 0.72 | | 0.68 | | | 0.63 | 0.74 | | |  |
| 36.9 – 37.2 | 0.75 | | 0.69 | | 0.81 | | 0.75 | | | 0.69 | 0.82 | | |  |
| > 37.2 | 1.30 | | 1.21 | | 1.39 | | 1.23 | | | 1.15 | 1.33 | | |  |
| *P value for linear trend* | <.0001 | |  | |  | | <.0001 | | |  |  | | |  |
| Oxygen saturation (SpO2), % |  | |  | |  | |  | | |  |  | | |  |
| < 90 | 1.97 | | 1.81 | | 2.14 | | 1.72 | | | 1.56 | 1.89 | | |  |
| Quintiles |  | |  | |  | |  | | |  |  | | |  |
| < 92 | 1.00 | | Reference | | | | 1.00 | | | Reference | | | |  |
| 92 – 94 | 0.54 | | 0.49 | | 0.60 | | 0.59 | | | 0.53 | 0.65 | | |  |
| 94 – 96 | 0.56 | | 0.51 | | 0.61 | | 0.61 | | | 0.55 | 0.67 | | |  |
| 96 – 98 | 0.66 | | 0.60 | | 0.71 | | 0.67 | | | 0.61 | 0.73 | | |  |
| > 98 | 0.61 | | 0.56 | | 0.66 | | 0.82 | | | 0.74 | 0.90 | | |  |
| *P value for linear trend* | <.0001 | |  | |  | | <.0001 | | |  |  | | |  |
| Platelet count x 10^9^ per L |  | |  | |  | |  | | |  |  | | |  |
| < 150 | 1.35 | | 1.28 | | 1.43 | | 1.27 | | | 1.19 | 1.34 | | |  |
| Quintiles |  | |  | |  | |  | | |  |  | | |  |
| < 157 | 1.00 | | Reference | | | | 1.00 | | | Reference | | | |  |
| 157 - 202 | 0.69 | | 0.65 | | 0.74 | | 0.74 | | | 0.69 | 0.79 | | |  |
| 202 – 250 | 0.71 | | 0.66 | | 0.77 | | 0.78 | | | 0.73 | 0.84 | | |  |
| 250 – 320 | 0.79 | | 0.73 | | 0.84 | | 0.85 | | | 0.79 | 0.91 | | |  |
| > 320 | 0.91 | | 0.85 | | 0.97 | | 0.93 | | | 0.87 | 1.00 | | |  |
| *P value for linear trend* | 0.72 | |  | |  | | 0.41 | | |  |  | | |  |
| C-reactive protein, mg/L |  | |  | |  | |  | | |  |  | | |  |
| > 10 | 2.19 | | 2.00 | | 2.40 | | 1.97 | | | 1.79 | 2.16 | | |  |
| Quintiles |  | |  | |  | |  | | |  |  | | |  |
| < 16 | 1.00 | | Reference | | | | 1.00 | | | Reference | | | |  |
| 16 – 42.8 | 1.28 | | 1.16 | | 1.42 | | 1.22 | | | 1.10 | 1.35 | | |  |
| 42.8 – 81 | 1.64 | | 1.49 | | 1.81 | | 1.50 | | | 1.36 | 1.65 | | |  |
| 81 – 143 | 2.04 | | 1.86 | | 2.23 | | 1.81 | | | 1.64 | 1.99 | | |  |
| > 143 | 4.00 | | 3.67 | | 4.37 | | 3.52 | | | 3.21 | 3.85 | | |  |
| *P value for linear trend* | <.0001 | |  | |  | | <.0001 | | |  |  | | |  |
| Ferritin, ng/mL |  | |  | |  | |  | | |  |  | | |  |
| > 300 | 2.04 | | 1.92 | | 2.18 | | 1.76 | | | 1.64 | 1.88 | | |  |
| Quintiles |  | |  | |  | |  | | |  |  | | |  |
| <176 | 1.00 | | Reference | | | | 1.00 | | | Reference | | | |  |
| 176 – 363 | 1.52 | | 1.37 | | 1.68 | | 1.37 | | | 1.24 | 1.52 | | |  |
| 363 - 625 | 1.92 | | 1.74 | | 2.12 | | 1.67 | | | 1.51 | 1.85 | | |  |
| 625 – 1100 | 2.38 | | 2.16 | | 2.62 | | 2.01 | | | 1.82 | 2.22 | | |  |
| >1100 | 3.45 | | 3.14 | | 3.79 | | 2.85 | | | 2.59 | 3.15 | | |  |
| *P value for linear trend* | <.0001 | |  | |  | | <.0001 | | |  |  | | |  |
| Lactate dehydrogenase, U/L |  | |  | |  | |  | | |  |  | | |  |
| > 280 | 3.09 | | 2.91 | | 3.30 | | 3.01 | | | 2.82 | 3.22 | | |  |
| Quintiles |  | |  | |  | |  | | |  |  | | |  |
| < 215 | 1.00 | | Reference | | | | 1.00 | | | Reference | | | |  |
| 215 – 277 | 1.51 | | 1.35 | | 1.69 | | 1.47 | | | 1.31 | 1.65 | | |  |
| 277 - 349 | 2.19 | | 1.97 | | 2.44 | | 2.10 | | | 1.88 | 2.35 | | |  |
| 349 – 466 | 3.21 | | 2.90 | | 3.56 | | 3.11 | | | 2.80 | 3.46 | | |  |
| > 466 | 6.88 | | 6.23 | | 7.60 | | 6.84 | | | 6.16 | 7.58 | | |  |
| *P value for linear trend* | <.0001 | |  | |  | | <.0001 | | |  |  | | |  |
| D-Dimer, ng/mL |  | |  | |  | |  | | |  |  | | |  |
| > 250 | 3.05 | | 2.76 | | 3.38 | | 2.80 | | | 2.52 | 3.11 | | |  |
| Quintiles |  | |  | |  | |  | | |  |  | | |  |
| < 230 | 1.00 | | Reference | | | | 1.00 | | | Reference | | | |  |
| 230 - 370 | 1.42 | | 1.23 | | 1.63 | | 1.33 | | | 1.16 | 1.54 | | |  |
| 370 – 590 | 2.22 | | 1.95 | | 2.53 | | 2.14 | | | 1.87 | 2.45 | | |  |
| 590 – 1030 | 3.43 | | 3.02 | | 3.89 | | 3.29 | | | 2.89 | 3.75 | | |  |
| > 1030 | 6.22 | | 5.51 | | 7.03 | | 5.90 | | | 5.19 | 6.71 | | |  |
| *P value for linear trend* | <.0001 | |  | |  | | <.0001 | | |  |  | | |  |
| Fibrinogen, mg/dL |  | |  | |  | |  | | |  |  | | |  |
| > 400 | 0.98 | | 0.90 | | 1.06 | | 0.77 | | | 0.70 | 0.84 | | |  |
| Quintiles |  | |  | |  | |  | | |  |  | | |  |
| < 374 | 1.00 | | Reference | | | | 1.00 | | | Reference | | | |  |
| 374 – 479 | 0.63 | | 0.56 | | 0.70 | | 0.57 | | | 0.51 | 0.65 | | |  |
| 479 – 579 | 0.74 | | 0.66 | | 0.83 | | 0.62 | | | 0.55 | 0.70 | | |  |
| 579 - 700 | 0.92 | | 0.82 | | 1.02 | | 0.72 | | | 0.64 | 0.82 | | |  |
| > 700 | 1.30 | | 1.17 | | 1.44 | | 0.89 | | | 0.79 | 1.00 | | |  |
| *P value for linear trend* | <.0001 | |  | |  | | 0.92 | | |  |  | | |  |
| **Symptoms** |  | |  | |  | |  | | |  |  | | |  |
| Hypoxemia | 1.73 | | 1.65 | | 1.82 | | 1.44 | | | 1.37 | 1.52 | | |  |
| Fever | 1.64 | | 1.55 | | 1.73 | | 1.28 | | | 1.21 | 1.36 | | |  |
| Cough | 1.14 | | 1.07 | | 1.22 | | 0.80 | | | 0.74 | 0.85 | | |  |
| Nausea/Vomiting | 0.90 | | 0.82 | | 0.99 | | 0.88 | | | 0.80 | 0.97 | | |  |
| Malaise and fatigue | 1.70 | | 1.60 | | 1.80 | | 1.43 | | | 1.34 | 1.52 | | |  |
| Dyspnea or shortness of breath | 2.06 | | 1.97 | | 2.16 | | 1.68 | | | 1.59 | 1.76 | | |  |
| **Diagnoses** |  | |  | |  | |  | | |  |  | | |  |
| Acute respiratory failure | 10.17 | | 9.56 | | 10.81 | | 8.55 | | | 8.02 | 9.11 | | |  |
| Pneumonia | 4.57 | | 4.31 | | 4.85 | | 3.47 | | | 3.26 | 3.69 | | |  |
| Sepsis | 6.34 | | 6.04 | | 6.65 | | 5.26 | | | 5.00 | 5.53 | | |  |
| Coagulation defects or hemorrhagic conditions | 4.27 | | 3.98 | | 4.59 | | 3.87 | | | 3.59 | 4.18 | | |  |
| Arrhythmia | 3.56 | | 3.34 | | 3.80 | | 2.90 | | | 2.71 | 3.10 | | |  |
| Heart failure | 2.68 | | 2.54 | | 2.83 | | 2.40 | | | 2.26 | 2.54 | | |  |
| MI | 3.52 | | 3.27 | | 3.78 | | 2.94 | | | 2.72 | 3.17 | | |  |
| **Treatments** |  | |  | |  | |  | | |  |  | | |  |
| Chloroquine/Hydroxychloroquine | 2.71 | | 2.57 | | 2.86 | | 1.95 | | | 1.80 | 2.12 | | |  |
| lopinavir/Ritonavir | 3.91 | | 3.22 | | 4.75 | | 2.24 | | | 1.81 | 2.76 | | |  |
| Remdesivir | 1.66 | | 1.58 | | 1.75 | | 2.66 | | | 2.50 | 2.83 | | |  |
| Dexamethasone | 1.71 | | 1.63 | | 1.79 | | 3.09 | | | 2.90 | 3.28 | | |  |
| ACEs/ARBs | 1.11 | | 1.05 | | 1.17 | | 0.97 | | | 0.91 | 1.02 | | |  |
| Anticoagulants | 2.79 | | 2.59 | | 3.00 | | 2.01 | | | 1.86 | 2.17 | | |  |
| Immunosuppressants | 4.79 | | 4.44 | | 5.16 | | 4.02 | | | 3.71 | 4.36 | | |  |
| Antibacterials for systemic use | 3.86 | | 3.62 | | 4.11 | | 3.34 | | | 3.13 | 3.56 | | |  |
| Antivirals for systemic use | 2.33 | | 2.10 | | 2.58 | | 1.87 | | | 1.68 | 2.09 | | |  |
| Corticosteroids for systemic use | 3.05 | | 2.91 | | 3.20 | | 3.81 | | | 3.61 | 4.01 | | |  |
| Abbreviations: COPD, chronic obstructive pulmonary disease; ACE, angiotensin-converting enzyme; ARB, angiotensin II receptor blocker; NSAIDS, non-steroidal anti-inflammatory drugs; PPIs, proton-pump inhibitors | | | | | | | | | | | | | | |
| *Adjusted for age, gender, region, race, and week of cohort entry. | | | | | |  | |  |  | | |  | | |
| **Includes acute and chronic kidney disease. | |  | |  | |  | |  |  | | |  | | |

| **Supplemental Table 3. Association of Baseline Characteristics and Clinical Variables During Hospitalization with Death among Hospitalized COVID-19 Patients** | | | | | | | | | | | | |  |  |
| --- | --- | --- | --- | --- | --- | --- | --- | --- | --- | --- | --- | --- | --- | --- |
|  | **Unadjusted Model** | | | | | | **Adjusted Model*** | | | | | | |  |
|  | **OR** | | **Lower Bound** | | **Upper Bound** | | **OR** | | | **Lower Bound** | **Upper Bound** | | |  |
| *Baseline* |  | |  | |  | |  | | |  |  | | |  |
| **Age** |  | |  | |  | |  | | |  |  | | |  |
| <10 | 0.20 | | 0.10 | | 0.43 | | 0.20 | | | 0.10 | 0.43 | | |  |
| 10-19 | 0.02 | | 0.00 | | 0.12 | | 0.02 | | | 0.00 | 0.14 | | |  |
| 20-29 | 0.12 | | 0.08 | | 0.16 | | 0.14 | | | 0.10 | 0.20 | | |  |
| 30-39 | 0.25 | | 0.20 | | 0.30 | | 0.27 | | | 0.22 | 0.33 | | |  |
| 40-49 | 0.58 | | 0.50 | | 0.66 | | 0.57 | | | 0.49 | 0.65 | | |  |
| 50-59 | 1.00 | | Reference | | | | 1.00 | | | Reference | | | |  |
| 60-69 | 1.84 | | 1.68 | | 2.01 | | 1.90 | | | 1.73 | 2.08 | | |  |
| 70-79 | 3.14 | | 2.88 | | 3.43 | | 3.48 | | | 3.18 | 3.81 | | |  |
| 80+ | 6.26 | | 5.75 | | 6.81 | | 7.61 | | | 6.96 | 8.32 | | |  |
| **Gender** |  | |  | |  | |  | | |  |  | | |  |
| Female | 0.69 | | 0.66 | | 0.72 | | 0.70 | | | 0.66 | 0.73 | | |  |
| Male | 1.00 | | Reference | | | | 1.00 | | | Reference | | | |  |
| **Race** |  | |  | |  | |  | | |  |  | | |  |
| African American | 0.76 | | 0.72 | | 0.81 | | 0.83 | | | 0.77 | 0.88 | | |  |
| Asian | 0.92 | | 0.79 | | 1.07 | | 0.94 | | | 0.80 | 1.12 | | |  |
| Caucasian | 1.00 | | Reference | | | | 1.00 | | | Reference | | | |  |
| Other/Unknown | 0.69 | | 0.65 | | 0.74 | | 1.10 | | | 1.01 | 1.20 | | |  |
| **Ethnicity** |  | |  | |  | |  | | |  |  | | |  |
| Hispanic | 0.55 | | 0.51 | | 0.60 | | 0.83 | | | 0.75 | 0.91 | | |  |
| Not Hispanic | 1.00 | | Reference | | | | 1.00 | | | Reference | | | |  |
| Unknown | 0.87 | | 0.80 | | 0.94 | | 1.10 | | | 1.00 | 1.21 | | |  |
| **Comorbidities** |  | |  | |  | |  | | |  |  | | |  |
| Diabetes | 1.71 | | 1.63 | | 1.79 | | 1.32 | | | 1.25 | 1.39 | | |  |
| Obesity | 0.90 | | 0.85 | | 0.95 | | 1.19 | | | 1.12 | 1.27 | | |  |
| Pulmonary disease |  | |  | |  | |  | | |  |  | | |  |
| COPD | 2.17 | | 2.04 | | 2.32 | | 1.37 | | | 1.28 | 1.47 | | |  |
| Asthma | 0.63 | | 0.57 | | 0.69 | | 0.87 | | | 0.79 | 0.97 | | |  |
| Cardiovascular disease |  | |  | |  | |  | | |  |  | | |  |
| Hypertension | 1.37 | | 1.30 | | 1.43 | | 0.85 | | | 0.81 | 0.89 | | |  |
| Coronary artery disease | 2.42 | | 2.29 | | 2.56 | | 1.23 | | | 1.16 | 1.31 | | |  |
| Congestive heart failure | 3.11 | | 2.94 | | 3.29 | | 1.82 | | | 1.71 | 1.94 | | |  |
| Kidney disease** | 4.66 | | 4.44 | | 4.89 | | 2.92 | | | 2.77 | 3.08 | | |  |
| Liver disease | 2.03 | | 1.85 | | 2.22 | | 2.56 | | | 2.31 | 2.83 | | |  |
| Cancer | 1.85 | | 1.72 | | 2.00 | | 1.58 | | | 1.45 | 1.71 | | |  |
| **Patient-reported medications** |  | |  | |  | |  | | |  |  | | |  |
| Statins | 1.40 | | 1.33 | | 1.47 | | 0.89 | | | 0.84 | 0.94 | | |  |
| ACEs/ARBs | 1.14 | | 1.08 | | 1.20 | | 0.83 | | | 0.78 | 0.88 | | |  |
| NSAIDS | 0.64 | | 0.58 | | 0.70 | | 0.84 | | | 0.76 | 0.92 | | |  |
| Steroids | 0.83 | | 0.77 | | 0.89 | | 1.00 | | | 0.92 | 1.09 | | |  |
| PPIs | 1.26 | | 1.19 | | 1.34 | | 1.04 | | | 0.97 | 1.10 | | |  |
| *During Hospitalization* |  | |  | |  | |  | | |  |  | | |  |
| **Observations (median, IQR)** |  | |  | |  | |  | | |  |  | | |  |
| Temperature, °C |  | |  | |  | |  | | |  |  | | |  |
| > 38 | 2.25 | | 2.08 | | 2.43 | | 2.34 | | | 2.14 | 2.56 | | |  |
| Quintiles |  | |  | |  | |  | | |  |  | | |  |
| < 36.4 | 1.00 | | Reference | | | | 1.00 | | | Reference | | | |  |
| 36.4 – 36.7 | 0.57 | | 0.53 | | 0.61 | | 0.55 | | | 0.50 | 0.59 | | |  |
| 36.7 – 36.9 | 0.49 | | 0.46 | | 0.54 | | 0.51 | | | 0.47 | 0.56 | | |  |
| 36.9 – 37.2 | 0.66 | | 0.61 | | 0.71 | | 0.68 | | | 0.62 | 0.74 | | |  |
| > 37.2 | 1.33 | | 1.24 | | 1.43 | | 1.37 | | | 1.27 | 1.48 | | |  |
| *P value for linear trend* | <.0001 | |  | |  | | <.0001 | | |  |  | | |  |
| Oxygen saturation (SpO2), % |  | |  | |  | |  | | |  |  | | |  |
| < 90 | 2.57 | | 2.36 | | 2.80 | | 2.63 | | | 2.38 | 2.90 | | |  |
| Quintiles |  | |  | |  | |  | | |  |  | | |  |
| < 92 | 1.00 | | Reference | | | | 1.00 | | | Reference | | | |  |
| 92 – 94 | 0.49 | | 0.44 | | 0.54 | | 0.49 | | | 0.44 | 0.55 | | |  |
| 94 – 96 | 0.45 | | 0.41 | | 0.49 | | 0.43 | | | 0.39 | 0.47 | | |  |
| 96 – 98 | 0.46 | | 0.42 | | 0.50 | | 0.42 | | | 0.38 | 0.47 | | |  |
| > 98 | 0.45 | | 0.41 | | 0.49 | | 0.51 | | | 0.47 | 0.57 | | |  |
| *P value for linear trend* | <.0001 | |  | |  | | <.0001 | | |  |  | | |  |
| Platelet count x 10^9^ per L |  | |  | |  | |  | | |  |  | | |  |
| < 150 | 2.21 | | 2.09 | | 2.33 | | 1.88 | | | 1.77 | 2.00 | | |  |
| Quintiles |  | |  | |  | |  | | |  |  | | |  |
| < 157 | 1.00 | | Reference | | | | 1.00 | | | Reference | | | |  |
| 157 - 202 | 0.55 | | 0.51 | | 0.59 | | 0.60 | | | 0.56 | 0.65 | | |  |
| 202 – 250 | 0.49 | | 0.46 | | 0.53 | | 0.58 | | | 0.53 | 0.62 | | |  |
| 250 – 320 | 0.46 | | 0.43 | | 0.49 | | 0.55 | | | 0.51 | 0.60 | | |  |
| > 320 | 0.41 | | 0.38 | | 0.44 | | 0.49 | | | 0.45 | 0.53 | | |  |
| *P value for linear trend* | <.0001 | |  | |  | | <.0001 | | |  |  | | |  |
| C-reactive protein, mg/L |  | |  | |  | |  | | |  |  | | |  |
| > 10 | 3.01 | | 2.70 | | 3.36 | | 2.61 | | | 2.32 | 2.93 | | |  |
| Quintiles |  | |  | |  | |  | | |  |  | | |  |
| < 16 | 1.00 | | Reference | | | | 1.00 | | | Reference | | | |  |
| 16 – 42.8 | 1.52 | | 1.35 | | 1.71 | | 1.41 | | | 1.25 | 1.59 | | |  |
| 42.8 – 81 | 2.20 | | 1.97 | | 2.46 | | 1.91 | | | 1.70 | 2.15 | | |  |
| 81 – 143 | 3.26 | | 2.94 | | 3.62 | | 2.83 | | | 2.53 | 3.17 | | |  |
| > 143 | 5.71 | | 5.16 | | 6.32 | | 5.31 | | | 4.76 | 5.92 | | |  |
| *P value for linear trend* | <.0001 | |  | |  | | <.0001 | | |  |  | | |  |
| Ferritin, ng/mL |  | |  | |  | |  | | |  |  | | |  |
| > 300 | 2.04 | | 1.91 | | 2.19 | | 1.91 | | | 1.77 | 2.06 | | |  |
| Quintiles |  | |  | |  | |  | | |  |  | | |  |
| <176 | 1.00 | | Reference | | | | 1.00 | | | Reference | | | |  |
| 176 – 363 | 1.58 | | 1.41 | | 1.76 | | 1.39 | | | 1.24 | 1.57 | | |  |
| 363 - 625 | 1.84 | | 1.65 | | 2.05 | | 1.61 | | | 1.44 | 1.81 | | |  |
| 625 – 1100 | 2.40 | | 2.16 | | 2.66 | | 2.24 | | | 2.00 | 2.51 | | |  |
| >1100 | 3.54 | | 3.21 | | 3.92 | | 3.39 | | | 3.03 | 3.79 | | |  |
| *P value for linear trend* | <.0001 | |  | |  | | <.0001 | | |  |  | | |  |
| Lactate dehydrogenase, U/L |  | |  | |  | |  | | |  |  | | |  |
| > 280 | 2.52 | | 2.36 | | 2.69 | | 2.81 | | | 2.61 | 3.02 | | |  |
| Quintiles |  | |  | |  | |  | | |  |  | | |  |
| < 215 | 1.00 | | Reference | | | | 1.00 | | | Reference | | | |  |
| 215 – 277 | 1.36 | | 1.21 | | 1.52 | | 1.38 | | | 1.22 | 1.56 | | |  |
| 277 - 349 | 1.56 | | 1.40 | | 1.74 | | 1.65 | | | 1.46 | 1.86 | | |  |
| 349 – 466 | 2.43 | | 2.19 | | 2.70 | | 2.73 | | | 2.43 | 3.06 | | |  |
| > 466 | 5.32 | | 4.82 | | 5.88 | | 6.97 | | | 6.23 | 7.79 | | |  |
| *P value for linear trend* | <.0001 | |  | |  | | <.0001 | | |  |  | | |  |
| D-Dimer, ng/mL |  | |  | |  | |  | | |  |  | | |  |
| > 250 | 4.34 | | 3.84 | | 4.91 | | 2.95 | | | 2.59 | 3.35 | | |  |
| Quintiles |  | |  | |  | |  | | |  |  | | |  |
| < 230 | 1.00 | | Reference | | | | 1.00 | | | Reference | | | |  |
| 230 - 370 | 1.72 | | 1.45 | | 2.03 | | 1.36 | | | 1.15 | 1.62 | | |  |
| 370 – 590 | 2.90 | | 2.48 | | 3.38 | | 2.05 | | | 1.74 | 2.41 | | |  |
| 590 – 1030 | 4.75 | | 4.09 | | 5.51 | | 3.03 | | | 2.59 | 3.54 | | |  |
| > 1030 | 9.35 | | 8.09 | | 10.79 | | 6.07 | | | 5.21 | 7.08 | | |  |
| *P value for linear trend* | <.0001 | |  | |  | | <.0001 | | |  |  | | |  |
| Fibrinogen, mg/dL |  | |  | |  | |  | | |  |  | | |  |
| > 400 | 0.92 | | 0.84 | | 1.00 | | 0.76 | | | 0.69 | 0.84 | | |  |
| Quintiles |  | |  | |  | |  | | |  |  | | |  |
| < 374 | 1.00 | | Reference | | | | 1.00 | | | Reference | | | |  |
| 374 – 479 | 0.73 | | 0.65 | | 0.83 | | 0.63 | | | 0.55 | 0.72 | | |  |
| 479 – 579 | 0.80 | | 0.71 | | 0.90 | | 0.67 | | | 0.59 | 0.77 | | |  |
| 579 - 700 | 0.80 | | 0.71 | | 0.90 | | 0.64 | | | 0.56 | 0.73 | | |  |
| > 700 | 1.11 | | 0.99 | | 1.25 | | 0.86 | | | 0.76 | 0.98 | | |  |
| *P value for linear trend* | 0.02 | |  | |  | | 0.09 | | |  |  | | |  |
| **Symptoms** |  | |  | |  | |  | | |  |  | | |  |
| Hypoxemia | 1.42 | | 1.35 | | 1.50 | | 1.16 | | | 1.10 | 1.22 | | |  |
| Fever | 1.30 | | 1.22 | | 1.38 | | 1.05 | | | 0.98 | 1.12 | | |  |
| Cough | 0.95 | | 0.88 | | 1.02 | | 0.70 | | | 0.65 | 0.76 | | |  |
| Nausea/Vomiting | 0.57 | | 0.50 | | 0.63 | | 0.62 | | | 0.55 | 0.70 | | |  |
| Malaise and fatigue | 1.49 | | 1.40 | | 1.59 | | 0.89 | | | 0.84 | 0.96 | | |  |
| Dyspnea or shortness of breath | 1.46 | | 1.39 | | 1.53 | | 1.18 | | | 1.12 | 1.25 | | |  |
| **Diagnoses** |  | |  | |  | |  | | |  |  | | |  |
| Acute respiratory failure | 3.98 | | 3.79 | | 4.19 | | 3.36 | | | 3.17 | 3.55 | | |  |
| Pneumonia | 2.58 | | 2.44 | | 2.72 | | 1.91 | | | 1.80 | 2.03 | | |  |
| Sepsis | 4.51 | | 4.30 | | 4.74 | | 4.14 | | | 3.92 | 4.38 | | |  |
| Coagulation defects or hemorrhagic conditions | 2.86 | | 2.65 | | 3.08 | | 2.48 | | | 2.27 | 2.70 | | |  |
| Arrhythmia | 2.73 | | 2.55 | | 2.91 | | 1.67 | | | 1.55 | 1.80 | | |  |
| Heart failure | 3.14 | | 2.97 | | 3.32 | | 1.85 | | | 1.74 | 1.96 | | |  |
| MI | 3.64 | | 3.38 | | 3.92 | | 2.29 | | | 2.11 | 2.49 | | |  |
| **Treatments** |  | |  | |  | |  | | |  |  | | |  |
| Chloroquine/Hydroxychloroquine | 1.99 | | 1.89 | | 2.11 | | 1.17 | | | 1.07 | 1.27 | | |  |
| lopinavir/Ritonavir | 3.67 | | 3.02 | | 4.46 | | 2.16 | | | 1.73 | 2.71 | | |  |
| Remdesivir | 0.97 | | 0.92 | | 1.02 | | 1.62 | | | 1.52 | 1.74 | | |  |
| Dexamethasone | 1.01 | | 0.96 | | 1.07 | | 1.71 | | | 1.61 | 1.83 | | |  |
| ACEs/ARBs | 0.78 | | 0.73 | | 0.82 | | 0.57 | | | 0.54 | 0.61 | | |  |
| Anticoagulants | 1.71 | | 1.60 | | 1.83 | | 1.01 | | | 0.94 | 1.09 | | |  |
| Immunosuppressants | 2.16 | | 1.99 | | 2.36 | | 2.20 | | | 2.00 | 2.42 | | |  |
| Antibacterials for systemic use | 2.50 | | 2.36 | | 2.65 | | 2.17 | | | 2.03 | 2.31 | | |  |
| Antivirals for systemic use | 1.79 | | 1.60 | | 1.99 | | 1.68 | | | 1.48 | 1.90 | | |  |
| Corticosteroids for systemic use | 1.49 | | 1.42 | | 1.56 | | 2.07 | | | 1.96 | 2.18 | | |  |
| Abbreviations: COPD, chronic obstructive pulmonary disease; ACE, angiotensin-converting enzyme; ARB, angiotensin II receptor blocker; NSAIDS, non-steroidal anti-inflammatory drugs; PPIs, proton-pump inhibitors | | | | | | | | | | | | | | |
| *Adjusted for age, gender, region, race, and week of cohort entry. | | | | | |  | |  |  | | |  | | |
| **Includes acute and chronic kidney disease. | |  | |  | |  | |  |  | | |  | | |
